# Spatial Localization of Defects in Halide Perovskites Using Photothermal Deflection Spectroscopy

**DOI:** 10.1021/acs.jpclett.3c02966

**Published:** 2024-01-26

**Authors:** Ales Vlk, Zdenek Remes, Lucie Landova, Katarina Ridzonova, Robert Hlavac, Antonin Fejfar, Martin Ledinsky

**Affiliations:** Institute of Physics of the Czech Academy of Sciences, Cukrovarnicka 10, 16200 Prague, Czech Republic

## Abstract

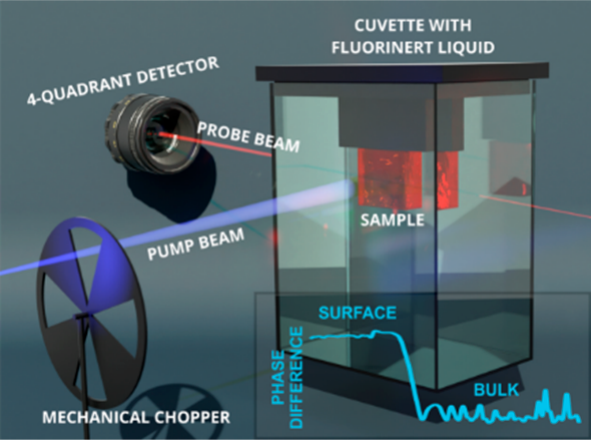

Photothermal deflection spectroscopy (PDS) emerges as a highly sensitive noncontact technique for measuring absorption spectra and serves for studying defect states within semiconductor thin films. In our study, we applied PDS to methylammonium lead bromide single crystals. By analyzing the frequency dependence of the PDS spectra and the phase difference of the signal, we can differentiate between surface and bulk deep defect absorption states. This methodology allowed us to investigate the effects of bismuth doping and light-induced degradation. The identified absorption states are attributed to MA^+^ vibrational states and structural defects, and their influence on the nonradiative recombination probability is discussed. This distinction significantly enhances our capability to characterize and analyze perovskite materials at a deeper level.

Absorption spectroscopy plays a crucial role in studying semiconductors for optoelectronic applications, particularly in materials such as metal halide perovskites (MHPs) used in photovoltaics. Through absorption spectra analysis, it is possible not only to predict the short-circuit current density (*J*_SC_) of the final photovoltaic (PV) device but also to identify absorption states within the optical gap and the open-circuit voltage (*V*_OC_) of the optimized device through Urbach energy.^1^ In MHP thin films, the defect states radiative emission probability, as well as defect absorption-induced photoconductivity, is low.^1^ This low activity poses challenges for traditional methods like photoluminescence spectroscopy (PL)^2^ and Fourier transform photocurrent spectroscopy (FTPS)^3^ in detecting these states deep within the bandgap. Therefore, a different approach is necessary to study deep defects in these materials.

Upon absorption of electromagnetic radiation, an electron–hole pair is created, and a considerable amount of photon energy is converted into heat through a thermalization process. The electron hole pair can undergo either radiative or nonradiative recombination. In nonradiative recombination, all the energy from the electron–hole pair is transformed into heat. In the case of radiative recombination, the resulting photon can either escape the perovskite film or be absorbed again, repeating the described process.^4^ Therefore, the direct detection of heat changes by photothermal deflection spectroscopy (PDS) may reveal more details mainly about deep defect absorption states. PDS is a pump and probe lock-in-based technique which detects changes in the sample’s temperature after absorption of the excitation radiation. Therefore, this method is highly sensitive to all absorption states which serve as nonradiative recombination centers. This makes it universal and suitable for all absorbing samples and a great choice to study deep defects in MHPs. Principles of PDS were first described by Jackson, Fournier, and Boccara.^5−7^ An intensity-modulated beam of monochromatic light (pump beam) is absorbed by the studied sample, causing transitions of electrons into excited states. Subsequent relaxation of the system via nonradiative transitions generates a time-dependent temperature change in the studied material and the surrounding liquid. This change is accompanied by a change in the index-of-refraction (IOR) of the surrounding liquid and the sample itself. In practice, the change of the IOR is studied by measuring the deflection of the probe laser beam using a position-sensitive 4-quadrant detector. The amplitude of the deflection is proportional to the optical absorption of the sample. Together with the probe beam deflection, a phase difference between Δφ the pump beam and the detected signal is measured as well.

According to the mutual position of the pump and probe beam, we distinguish two configurations: (1) collinear c-PDS and (2) transverse t-PDS (see Figure S1 in the Supporting Information).^5^ In both configurations, the pump beam is perpendicular to the sample surface. In the collinear variant of PDS, both the pump and probe beam are quasi-parallel to each other. The deviation of the probe beam directly reflects the change of the IOR of the sample. In this configuration, the probe beam can be neither absorbed nor scattered by the sample. Also due to this fact, we use the transverse configuration in our setup. Here, the sample is immersed in a chemically inert, transparent liquid. The probe beam is parallel to the surface of the sample, probing the change of the index of refraction of the liquid in the proximity of the sample surface. In t-PDS, the signal-to-noise ratio can be improved by choosing liquid with a stronger dependence of IOR on temperature, which leads to a significant deflection of the probe beam (in our setup, we use Fluorinert FC72).

PDS is widely used for the characterization of almost any thin film.^3,8−13^ The most important prerequisite for this measurement is a suitable substrate. It should be nonabsorbing in the area of interest and, moreover, have very low heat conductivity. The second requirement limits heat dissipation into the substrate and thus maximizes the PDS sensitivity. Quartz substrates are the most frequently used to this end.

In this paper, our objective is to expand the application of PDS to perovskite single crystals. Through detailed numerical simulations, we gained a comprehensive understanding of the measured results. Our findings demonstrate that the frequency dependence of PDS and the phase difference can effectively differentiate between deep defects located at the sample surface and those within the bulk of the material.

For the PDS measurement interpretation, the absorption length *l*_α_ of the excitation radiation and thermal diffusion length (sometimes termed thermal length) μ_t_ in the sample are the most important parameters. The absorption depth is defined as the reciprocal value of the absorption coefficient α [cm^–1^], i.e., *l*_α_ = 1/α, and it defines the volume where the heat is generated in the probed sample. Depending on the values of *l*_α_, we can distinguish two main regimes of heat generation in the measured sample of a thickness *D*. First, strong absorption *l*_α_ < *D*, where all photons are absorbed in the sample. The second regime of weak absorption *l*_α_ > *D* describes the situation when the light is only partially absorbed in the sample and most of the light will be transmitted.

The thermal diffusion length μ_t_ defines the characteristic length scale (distance over which) the heat change in the material propagates. It is inversely proportional to the thermal diffusion coefficient, and it depends on the pump beam modulation (chopping) frequency ω [Hz] (see eq 1).^14^
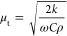
1where ρ [kg m^–3^] is the sample density, *C* [J kg^–1^ K^–1^] the specific heat capacity, and *k* [W m^–1^ K^–1^] the thermal conductivity. Larger values of μ_t_ mean that the heat can propagate to the detection point from a greater distance.

In the case of μ_t_ > *D*, which is typical for most bulk crystals, the high thermal conductivity of the crystalline lattice or free charge carriers (in the case of semiconductors with a high diffusion length of charge carriers) redistributes the heat across the whole sample. The thermal contrast on the measured front surface is in such a case insufficient for detection, mainly at the low absorption region in the semiconductor bandgap. That is why the PDS in transverse configuration is now used exclusively for absorption measurement of thin films (TF) deposited on low thermally conductive and nonabsorbing substrates, most often quartz. Here, the substrates’ thermal diffusion length μ_tS_ limits the heat transfer on the back surface, which results in sufficient signal collection efficiency and measurement sensitivity of absorption down to 10^–4^. Indeed, the low heat conductivity is a fundamental prerequisite for PDS measurements of bulk samples. Halide perovskites have low thermal conductivity (*k* ≈ 0.37–0.51 W m^–1^ K^–1^);^15−17^ therefore, the thermal redistribution is limited to the volume near the front surface, and the heat changes may be effectively detected. In fact, the methylammonium lead bromide (MAPbBr_3_) single crystal has a lower heat conductivity than the quartz substrate used for thin films’ PDS measurement. Therefore, the PDS measurement on MHP single crystals has the potential for high detectivity even below 10^–4^. Once the necessary condition μ_t_ < *D* is fulfilled, we have to discuss the relationship between heat generation depth and thermal diffusion length (*l*_α_ and μ_t_):

In the situation of high absorption of the excitation light, where *l*_α_ < μ_t_, all the incident photons are absorbed within μ_t_, which also limits the probed volume. The heat is thus effectively transferred to the detection/cooling medium and detected by the probe beam. Therefore, the total detected signal does not change for strongly absorbed photons above the bandgap, even if the absorption coefficient α is not constant. That leads to saturation of the PDS signal; see Figure 1a for energies above 2.4 eV.

**Figure 1:**
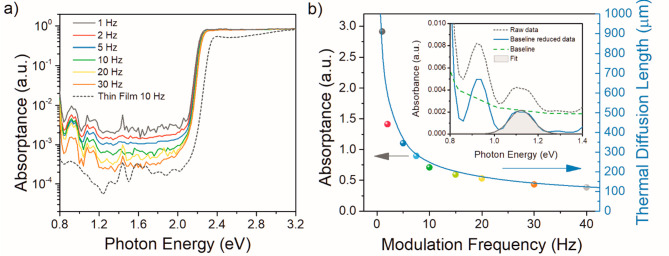
(a) Frequency dependence of the normalized absorption spectra of MAPbBr_3_ single crystal of dimensions 5 × 5 × 2 mm and reference spectra of MAPbBr_3_ thin film determined using the PDS method. (b) Comparison of absorptance of MAPbBr_3_ SC determined from the frequency dependence and thermal diffusion length dependence on modulation frequency. The inset of (b) shows the part of the PDS spectra, from which the absorptance values at given frequency were taken.

For the case of weakly absorbed light, when *l*_α_ > μ_t_, any change of the absorption coefficient α results in a change of the heat generated within the probed volume μ_t_, and thus, the detected PDS signal changes. For the MAPbBr_3_ single crystal shown in Figure 1a, we can see that this regime occurs below 2.2 eV.

As previously mentioned, thermal diffusion length μ_t_ limits the probed volume. Therefore, according to eq 1, the detection volume is frequency-dependent. Comparison of μ_t_ of different perovskite materials calculated for ω = 10 Hz is in Table S1. From eq 1, we can see that with decreasing frequency, the probed volume increases. In other words, for lower frequency, the heat has more time to propagate from larger depths toward the surface and be detected. For a given absorption coefficient, the probed volume can be increased by decreasing the frequency. A larger probed volume results in a larger contribution of bulk states to the detected signal. However, the amount of signal generated by absorption directly at the surface defect states will not be affected. Therefore, we can use the frequency dependence of normalized PDS signal to distinguish between bulk and surface sub-bandgap electronic states (defects/impurities). Additionally, a larger probed volume of the bulk crystal in comparison with thin film results in a better signal-to-noise ratio and, thus, better sensitivity in the weak absorption region below the absorption edge. Moreover, in Figure 1a, we observe a red-shift of the absorption edge with a decrease in modulation frequency. This shift is caused by an extension of the saturated region due to an increase of μ_t_, i.e., the condition *l*_α_ < μ_t_ is valid for smaller absorption coefficients.

As a result of the better signal-to-noise ratio of the measurement on the bulk MAPbBr_3_ SC in comparison with the thin film, we were able to observe two significant peaks at 0.925 and 1.110 eV. A part of a third peak below 0.80 eV appears as well at the edge of the detection range of our PDS setup. Figure 1b shows the frequency dependence of the integrated intensity of the peak located at 1.110 eV. In order to obtain the intensity, the baseline caused by free carrier absorption^12,18,19^ was removed, and the peak was fitted using the Gaussian function. Here, we can see that the absorptance decreases following the (1/ω)^1/2^ trend, which is in line with eq 1. The μ_t_ was calculated using parameters in Table S1. Because the obtained signal is proportional to the detection depth of PDS measurement, we can conclude that these deep bandgap absorption states are localized in the bulk of the perovskite SC.

In order to find the origin of these deep bandgap states, we performed PDS measurements on MAPbBr_3_, MAPbCl_3_, and CsPbBr_3_ SCs. As we can see in Figure 2, the replacement of the Br^–^ leads to a shift of the band edge but does not influence the position of the observed absorption peaks in the bandgap. On the other hand, these states completely disappear for fully inorganic CsPbBr_3_ SC, when the MA^+^ is replaced by inorganic cesium Cs^+^. That implies that these absorption states are directly associated with the internal vibrational states of MA^+^. Moreover, there is a constant energy difference of 0.185 eV between the detected peaks, which is approximately 1500 cm^–1^ and agrees with the energy of the vibrational modes of CH and NH bonds as measured by IR absorption spectroscopy.^20,21^ Therefore, the PDS detected absorption peaks energies agree with the multiphonon absorption processes—higher overtones of CH and NH bond vibrational modes located around 1500 and 3000 cm^–1^ (see Figure S4).^20,21^

**Figure 2:**
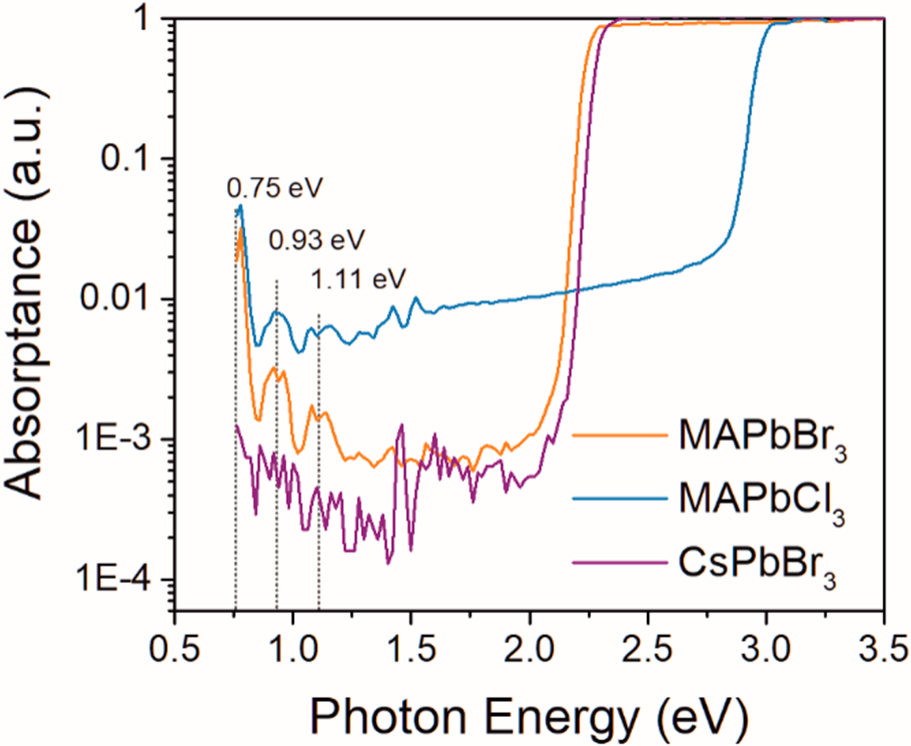
Comparison of PDS absorption spectra of MAPbBr_3_, MAPbCl_3_, and CsPbBr_3_ single crystals.

The MA^+^-related absorption states around the middle of the MAPbBr_3_ bandgap are potentially nonradiative recombination centers for charge carriers. But because these are multiphonon absorption states, the probability of the nonradiative recombination is very low, as described by Kirchartz et al.^22^ Therefore, the MA^+^-related states do not significantly affect the transport properties or the open circuit voltage of the finalized solar cell.

PDS is a lock-in-based technique; therefore, the phase difference Δφ between the pump beam and measured signal is detected. The heat generated directly at the surface is transferred immediately into the liquid and thus not retarded (Δφ = 0°) behind the pump beam. On the other hand, the heat generated in the bulk of the sample has to propagate toward the surface and then transfer into the liquid. This causes a time delay and thus increase in the phase shift (Δφ ∼ 0° for strong absorption; Δφ < 0° for weak absorption). This parameter can therefore provide additional information about the localization of the absorption states. To support previous statement and improve our understanding of the meaning of the Δφ, we used the finite element method to calculate the evolution of surface temperature during the periodic heating for different excitation energies (different absorption coefficients α) and compared it with our measurements (see Figures 3 and S5–S7). For strong absorption, the phase difference is set to 0° for both measured and calculated phase differences. In Figure 3, we can see that the calculated and measured |Δφ| at the absorption edge increases with a decrease in absorption coefficient. That means that the detected signal originates from greater depths and thus is more retarded behind the excitation. Good agreement between the measured and calculated data in the strong absorption region and the absorption edge validates our model description of the PDS experiment.

**Figure 3:**
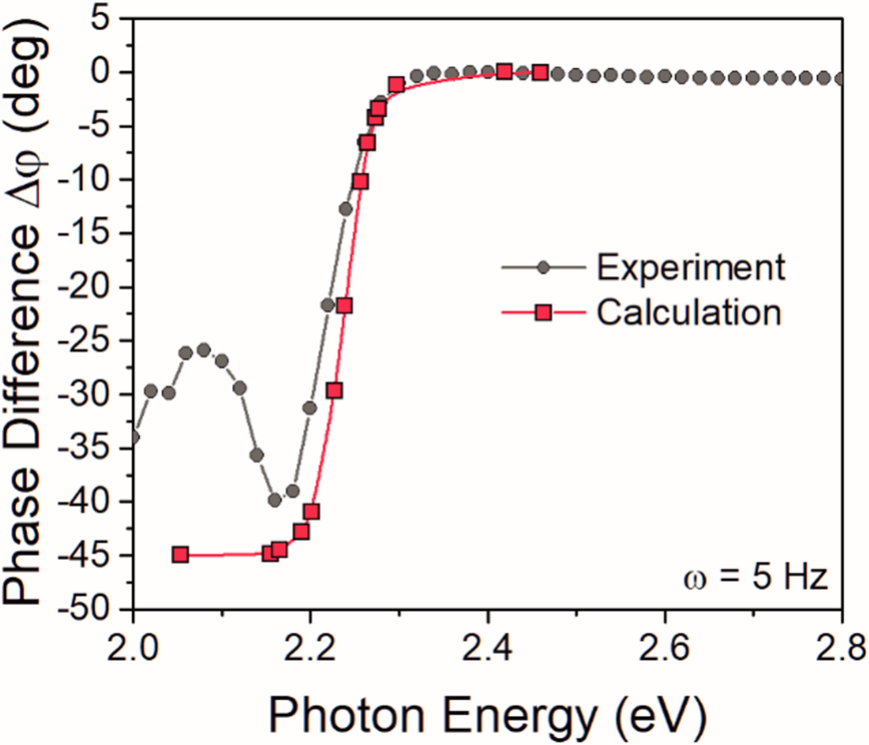
Comparison of experimentally measured and calculated phase difference Δφ of the PDS signal for a modulation frequency of 5 Hz.

But once the absorption depth is greater than the PDS detection volume (*l*_α_ > μ_t_), the signal delay is given by the μ_t_ only and should not change anymore with decreasing light energy, as we obtained for the calculated spectra. The phase difference at low energies should be constant, in this particular case at around 45°. However, the measurement shows a substantial return of phase shift to values around −25° in this low absorption spectral part. This inconsistency is given by the incompleteness of our numerical model, where we do not take into account the surface state defects/absorption. Additional absorption, which takes place directly at the surface, naturally leads to decreases in the PDS signal retardation. Therefore, by observing the behavior of the |Δφ| in the weak absorption regime, we can determine whether the observed absorption signal originates mostly from the bulk defect states or comes from the surface defects. In the following text, we demonstrate the use of phase difference analysis in two examples.

Figure 4 shows the evolution of absorption spectra and phase difference Δφ of 4% bismuth-doped MAPbBr_3_ SC. As can be seen, the addition of Bi into the perovskite lattice introduces sub-bandgap states between 1.2 and 1.9 eV in comparison with dopant-free SC.^23,24^ As in the case of pure MAPbBr_3_ SC, the sub-bandgap signal decreases with an increase in modulation frequency, and it is proportional to (1/ω)^1/2^, which confirms that the bismuth is uniformly distributed within the whole crystal volume. This is in agreement with the results of Abdelhady et al.^25^

**Figure 4:**
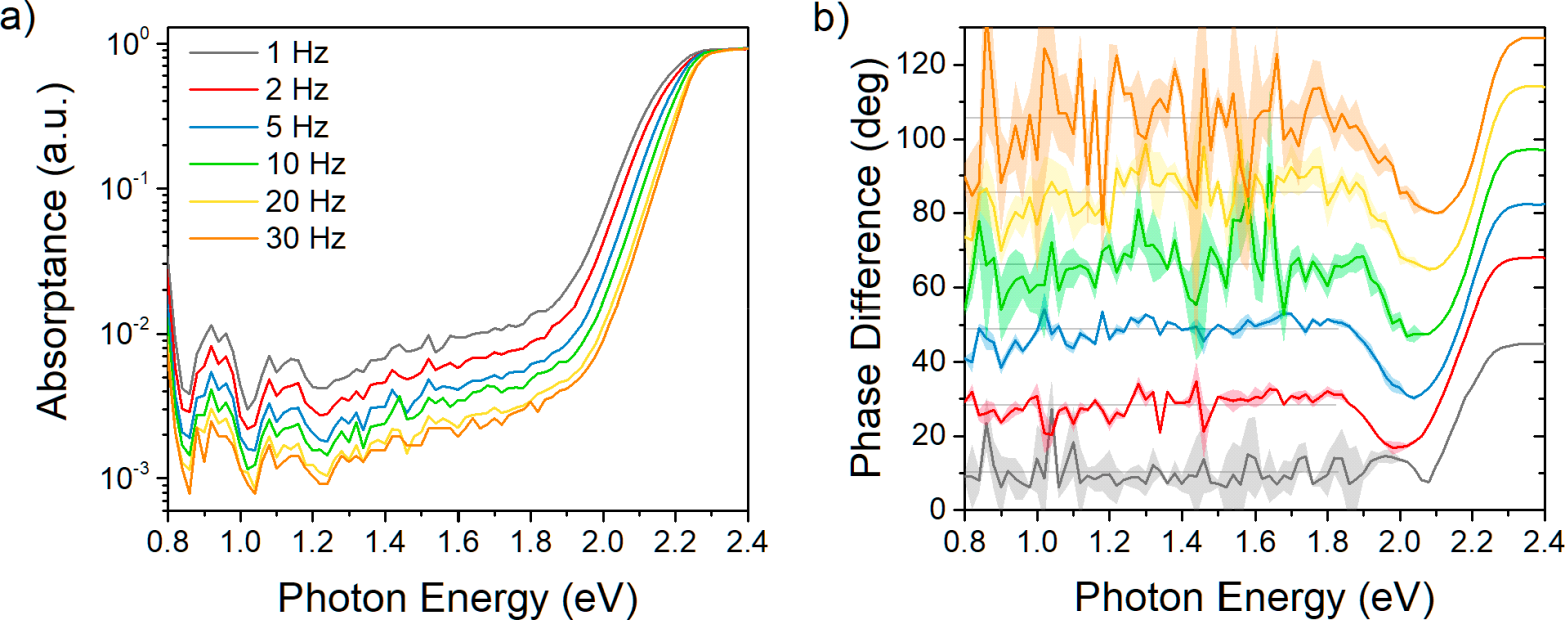
(a) Frequency dependence of the absorption spectra of bismuth-doped MAPbBr_3_ single crystal determined using the PDS method. (b) Stacked plot of frequency dependence of phase difference Δφ between pump and probe beam. The offset is introduced to make the data easier to read.

Because the change of the modulation frequency leads to a different PDS depth sensitivity, we are able in this way to change the ratio between surface and bulk contributing to the measured signal. For the modulation frequency of 1 Hz, the measurement is the most sensitive to the bulk properties of the sample. Below the absorption edge, where the probed depth is determined by the thermal diffusion length, a saturation of |Δφ| is observed (see Figure 4b), and we do not observe a decrease of the phase shift. Therefore, we conclude that the PDS signal originates from the bulk absorption states of the doped MAPbBr_3_ SC. The contribution of the surface states is negligible for this modulation frequency.

The use of higher modulation frequencies decreases the PDS detection depth, which leads to a high sensitivity to surface defect states. This can be clearly seen in the phase difference depicted in Figure 4b. As the modulation frequency increases, the |Δφ| in the sub-bandgap region returns closer to the strong absorption level (|Δφ| = 0°); the dip at 2.0 eV is more and more pronounced. As the probe volume of the sample reduces, the surface sensitivity of this measurement increases. This provides evidence that besides the bulk defects introduced into the sample by Bi doping there are high densities of surface defects as well. Similar behavior is observed for the undoped MAPbBr_3_ SC.

A different effect compared to the addition of Bi has the exposure of perovskite SC to light under an ambient atmosphere. Figure 5 shows the sub-bandgap absorption determined using PDS and the corresponding phase difference.

**Figure 5:**
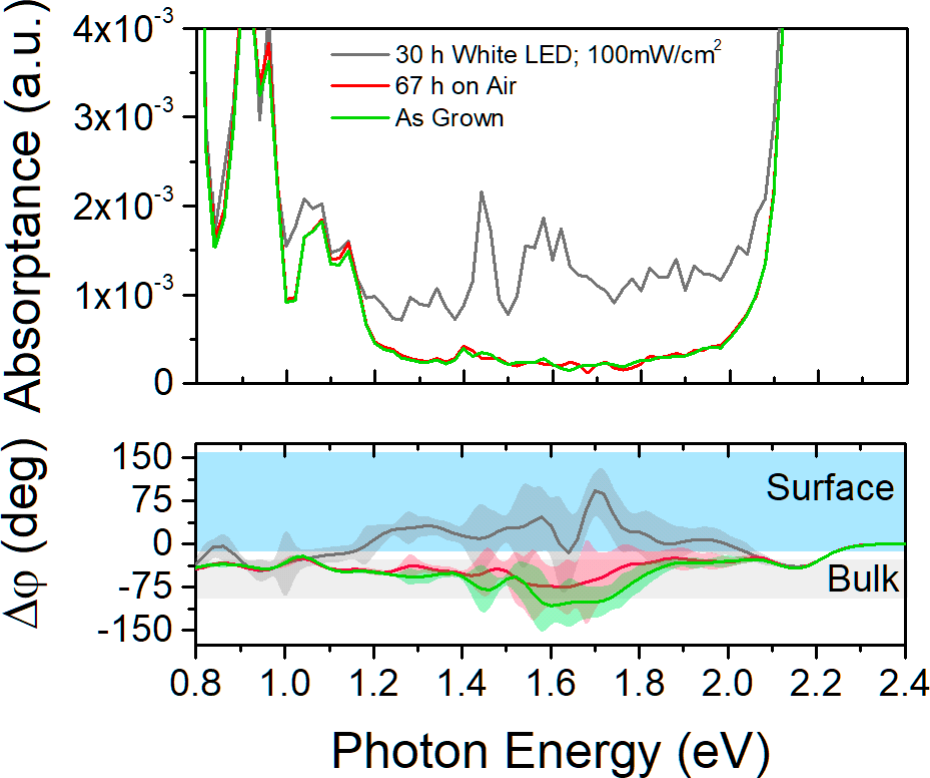
Absorption spectra and phase difference Δφ of MAPbBr_3_ SC (dimensions 6 mm × 6 mm × 2 mm) exposed to different surrounding conditions. The measurement was performed with a 6.6 Hz modulation frequency.

First, pure MAPbBr_3_ SC was measured without any exposure to the ambient atmosphere, i.e., the sample was inserted into the inert, nonpolar liquid used for PDS measurement inside the nitrogen glovebox. This prevents exposure to air and water and thus the degradation of the sample before or during the PDS measurement. As we can see in Figure 5, the absorption between 1.2 and 2.1 eV of the fresh sample is on the level of 2 × 10^–4^. The PDS phase difference |Δφ| significantly increases at energies below the absorption edge. This means that the detected signal is significantly retarded behind the pump beam, and thus its majority originates in the bulk of the sample. Exposure of the sample to an ambient atmosphere in the dark for 67 h had no impact on measured absorption spectra and minimal impact on the phase difference. However, additional illumination for 30 h with simultaneous exposure to ambient air significantly affected the absorption spectra of the sample. After the light exposure, an increase of absorption between 1.2 and 2.1 eV is observed, with two prominent bands at 1.45 and 1.60 eV. The same band at 1.45 eV was observed in Figure 1a. As can be seen, the |Δφ| in this energy interval decreases to the same value as that observed for strong absorption. This means that a majority of the electron–hole pairs recombine directly at the highly defective surface. Exposure to the light without encapsulation thus creates a large number of surface deep defects. Moreover, below 1.2 eV we can see that for all of the measurements, the |Δφ| returns to the same value for all measurements. This is caused by previously discussed MA^+^-related absorption states in the bulk of the single crystal.

In conclusion, we outline a way and necessary conditions for PDS absorption measurement on bulk samples; in particular, we present this measurement on halide perovskite single crystals. This approach provides a significant sensitivity gain in the low absorption region compared to standard PDS measurements conducted on thin films.

We observe states at 0.740, 0.925, and 1.110 eV, which are associated with internal vibrations of the MA cation in the perovskite cage. These states do not act as active recombination centers due to their low electron–multiphonon interaction cross section. Our study demonstrates that through the analysis of frequency-dependent PDS spectra and behavior of the phase difference between pump beam and detected signal, we are able to distinguish between bulk and surface absorption states. Using this approach, we provide direct evidence confirming the bulk character of the defect states induced by Bi doping. On the contrary, the defect states observed at 1.40 and 1.65 eV, induced by light exposure, are localized at the surface of the MAPbBr_3_ single crystal.

### Experimental Methods

For the PDS measurements, a home-built PDS setup was used. The scheme of the setup is shown in Figure S2. As a light source, we used a 150 W Xe lamp and a monochromator equipped with three gratings blazed at 300, 750, and 1250 nm operating over a broad spectral range from the ultraviolet to infrared region 250–1700 nm (≈ 0.73–5 eV). About 8% of light is reflected by the beamsplitter into the integrating sphere equipped with Si and InGaAs photodiodes. The spot size has dimensions of approximately 3 mm × 0.5 mm (width × height). The HeNe high point stability laser was used to detect the IOR changes in the detection/cooling media (FC72 fluorinert liquid) in the vicinity of the illuminated sample’s surface. The cuvette dimensions are 42.5 mm × 12.5 mm × 45.0 mm. As a reference/calibration sample we used thin layer of carbon nantotubes (see Figure S3).

Samples used in this study were prepared using procedures in respective publications: a thin film of MAPbBr_3_,^26^ single crystals of MAPbBr_3_ (frequency dependence: 5 × 5 × 2 mm^3^, degradation measurement: 6 × 6 × 2 mm^3^),^25^ bismuth-doped SC of MAPbBr_3_ (3 × 3 × 1.5 mm^3^),^25^ MAPbCl_3_ (3 × 3 × 1.5 mm^3^) single crystal,^27^ and CsPbBr_3_ (3 × 3 × 1.5 mm^3^) single crystal.^28^ The recipe is provided in the Supporting Information.

In order to obtain the error of the measurement, each point is measured several times (10×–20× based on signal strength). The median is taken from those values, and these are the data points. The measurement errors are represented by the standard deviation.

A brief description of the surface temperature simulation is given in the Supporting Information.

## Supporting Information Available

A description of sample preparation, PDS measurement setup, calculations of thermal diffusion lengths and surface temperatures (PDF)
Transparent Peer Review report available (PDF)

## References

[ref1] Ugur E.; Ledinský M.; Allen T. G.; Holovský J.; Vlk A.; De Wolf S. Life on the Urbach Edge. J. Phys. Chem. Lett. 2022, 13 (33), 7702–7711. 10.1021/acs.jpclett.2c01812. 35960888

[ref2] Ledinský M.; Vlk A.; Schönfeldová T.; Holovský J.; Aydin E.; Dang H. X.; Hájková Z.; Landová L.; Valenta J.; Fejfar A.; De Wolf S. Impact of Cation Multiplicity on Halide Perovskite Defect Densities and Solar Cell Voltages. J. Phys. Chem. C 2020, 124 (50), 27333–27339. 10.1021/acs.jpcc.0c08193.

[ref3] Holovský J.; De Wolf S.; Werner J.; Remeš Z.; Müller M.; Neykova N.; Ledinský M.; Černá L.; Hrzina P.; Löper P.; Niesen B.; Ballif C. Photocurrent Spectroscopy of Perovskite Layers and Solar Cells: A Sensitive Probe of Material Degradation. J. Phys. Chem. Lett. 2017, 8 (4), 838–843. 10.1021/acs.jpclett.6b02854. 28121155

[ref4] Sell J. Photothermal Investigations of Solids and Fluids; Elsevier Science: St. Louis, MO, 2014.

[ref5] Jackson W. B.; Amer N. M.; Boccara A. C.; Fournier D. Photothermal Deflection Spectroscopy and Detection. Appl. Opt. 1981, 20 (8), 1333 10.1364/AO.20.001333. 20309309

[ref6] Boccara A. C.; Jackson W.; Amer N. M.; Fournier D. Sensitive Photothermal Deflection Technique for Measuring Absorption in Optically Thin Media. Opt. Lett. 1980, 5 (9), 377 10.1364/OL.5.000377. 19693234

[ref7] Boccara A. C.; Fournier D.; Badoz J. Thermo-optical Spectroscopy: Detection by the ’’mirage Effect’’. Appl. Phys. Lett. 1980, 36 (2), 130–132. 10.1063/1.91395.

[ref8] De Wolf S.; Holovsky J.; Moon S.-J.; Löper P.; Niesen B.; Ledinsky M.; Haug F.-J.; Yum J.-H.; Ballif C. Organometallic Halide Perovskites: Sharp Optical Absorption Edge and Its Relation to Photovoltaic Performance. J. Phys. Chem. Lett. 2014, 5 (6), 1035–1039. 10.1021/jz500279b. 26270984

[ref9] Kiyek V. M.; Birkhölzer Y. A.; Smirnov Y.; Ledinsky M.; Remes Z.; Momand J.; Kooi B. J.; Koster G.; Rijnders G.; Morales-Masis M. Single-Source, Solvent-Free, Room Temperature Deposition of Black γ-CsSnI _3_ Films. Adv. Mater. Interfaces 2020, 7 (11), 2000162 10.1002/admi.202000162.

[ref10] Ugur E.; Alarousu E.; Khan J. I.; Vlk A.; Aydin E.; De Bastiani M.; Balawi A. H.; Gonzalez Lopez S. P.; Ledinský M.; De Wolf S.; Laquai F. How Humidity and Light Exposure Change the Photophysics of Metal Halide Perovskite Solar Cells. Sol. RRL 2020, 4 (11), 2000382 10.1002/solr.202000382.

[ref11] Nesládek M.; Vaněček M.; Rosa J.; Quaeyhaegens C.; Stals L. M. Subgap Optical Absorption in CVD Diamond Films Determined from Photothermal Deflection Spectroscopy. Diam. Relat. Mater. 1995, 4 (5–6), 697–701. 10.1016/0925-9635(94)05248-4.

[ref12] Remes Z.; Vanecek M.; Yates H. M.; Evans P.; Sheel D. W. Optical Properties of SnO_2_:F Films Deposited by Atmospheric Pressure CVD. Thin Solid Films 2009, 517 (23), 6287–6289. 10.1016/j.tsf.2009.02.109.

[ref13] Frye R. C.; Kumler J. J.; Wong C. C. Investigation of Surface Passivation of Amorphous Silicon Using Photothermal Deflection Spectroscopy. Appl. Phys. Lett. 1987, 50 (2), 101–103. 10.1063/1.97866.

[ref14] Rosencwaig A.; Gersho A. Theory of the Photoacoustic Effect with Solids. J. Appl. Phys. 1976, 47 (1), 64–69. 10.1063/1.322296.

[ref15] Ge C.; Hu M.; Wu P.; Tan Q.; Chen Z.; Wang Y.; Shi J.; Feng J. Ultralow Thermal Conductivity and Ultrahigh Thermal Expansion of Single-Crystal Organic–Inorganic Hybrid Perovskite CH _3_ NH _3_ PbX _3_ (X = Cl, Br, I). J. Phys. Chem. C 2018, 122 (28), 15973–15978. 10.1021/acs.jpcc.8b05919.

[ref16] Haeger T.; Heiderhoff R.; Riedl T. Thermal Properties of Metal-Halide Perovskites. J. Mater. Chem. C 2020, 8 (41), 14289–14311. 10.1039/D0TC03754K.

[ref17] Elbaz G. A.; Ong W.-L.; Doud E. A.; Kim P.; Paley D. W.; Roy X.; Malen J. A. Phonon Speed, Not Scattering, Differentiates Thermal Transport in Lead Halide Perovskites. Nano Lett. 2017, 17 (9), 5734–5739. 10.1021/acs.nanolett.7b02696. 28806090

[ref18] Fox M. Optical Properties of Solids; Oxford Master Series in Condensed Matter Physics; Oxford University Press: Oxford, 2001.

[ref19] Kucharski R.; Janicki Ł.; Zajac M.; Welna M.; Motyka M.; Skierbiszewski C.; Kudrawiec R. Transparency of Semi-Insulating, n-Type, and p-Type Ammonothermal GaN Substrates in the Near-Infrared, Mid-Infrared, and THz Spectral Range. Crystals 2017, 7 (7), 187 10.3390/cryst7070187.

[ref20] Glaser T.; Müller C.; Sendner M.; Krekeler C.; Semonin O. E.; Hull T. D.; Yaffe O.; Owen J. S.; Kowalsky W.; Pucci A.; Lovrinčić R. Infrared Spectroscopic Study of Vibrational Modes in Methylammonium Lead Halide Perovskites. J. Phys. Chem. Lett. 2015, 6 (15), 2913–2918. 10.1021/acs.jpclett.5b01309. 26267180

[ref21] Schuck G.; Többens D. M.; Koch-Müller M.; Efthimiopoulos I.; Schorr S. Infrared Spectroscopic Study of Vibrational Modes across the Orthorhombic–Tetragonal Phase Transition in Methylammonium Lead Halide Single Crystals. J. Phys. Chem. C 2018, 122 (10), 5227–5237. 10.1021/acs.jpcc.7b11499.

[ref22] Kirchartz T.; Markvart T.; Rau U.; Egger D. A. Impact of Small Phonon Energies on the Charge-Carrier Lifetimes in Metal-Halide Perovskites. J. Phys. Chem. Lett. 2018, 9 (5), 939–946. 10.1021/acs.jpclett.7b03414. 29409323

[ref23] Ulatowski A. M.; Wright A. D.; Wenger B.; Buizza L. R. V.; Motti S. G.; Eggimann H. J.; Savill K. J.; Borchert J.; Snaith H. J.; Johnston M. B.; Herz L. M. Charge-Carrier Trapping Dynamics in Bismuth-Doped Thin Films of MAPbBr _3_ Perovskite. J. Phys. Chem. Lett. 2020, 11 (9), 3681–3688. 10.1021/acs.jpclett.0c01048. 32302145

[ref24] Yavari M.; Ebadi F.; Meloni S.; Wang Z. S.; Yang T. C.-J.; Sun S.; Schwartz H.; Wang Z.; Niesen B.; Durantini J.; Rieder P.; Tvingstedt K.; Buonassisi T.; Choy W. C. H.; Filippetti A.; Dittrich T.; Olthof S.; Correa-Baena J.-P.; Tress W. How Far Does the Defect Tolerance of Lead-Halide Perovskites Range? The Example of Bi Impurities Introducing Efficient Recombination Centers. J. Mater. Chem. A 2019, 7 (41), 23838–23853. 10.1039/C9TA01744E.

[ref25] Abdelhady A. L.; Saidaminov M. I.; Murali B.; Adinolfi V.; Voznyy O.; Katsiev K.; Alarousu E.; Comin R.; Dursun I.; Sinatra L.; Sargent E. H.; Mohammed O. F.; Bakr O. M. Heterovalent Dopant Incorporation for Bandgap and Type Engineering of Perovskite Crystals. J. Phys. Chem. Lett. 2016, 7 (2), 295–301. 10.1021/acs.jpclett.5b02681. 26727130

[ref26] Holovský J.; Peter Amalathas A.; Landová L.; Dzurňák B.; Conrad B.; Ledinský M.; Hájková Z.; Pop-Georgievski O.; Svoboda J.; Yang T. C.-J.; Jeangros Q. Lead Halide Residue as a Source of Light-Induced Reversible Defects in Hybrid Perovskite Layers and Solar Cells. ACS Energy Lett. 2019, 4 (12), 3011–3017. 10.1021/acsenergylett.9b02080.

[ref27] Maculan G.; Sheikh A. D.; Abdelhady A. L.; Saidaminov M. I.; Haque M. A.; Murali B.; Alarousu E.; Mohammed O. F.; Wu T.; Bakr O. M. CH _3_ NH _3_ PbCl _3_ Single Crystals: Inverse Temperature Crystallization and Visible-Blind UV-Photodetector. J. Phys. Chem. Lett. 2015, 6 (19), 3781–3786. 10.1021/acs.jpclett.5b01666. 26722870

[ref28] Saidaminov M. I.; Haque M. A.; Almutlaq J.; Sarmah S.; Miao X.-H.; Begum R.; Zhumekenov A. A.; Dursun I.; Cho N.; Murali B.; Mohammed O. F.; Wu T.; Bakr O. M. Inorganic Lead Halide Perovskite Single Crystals: Phase-Selective Low-Temperature Growth, Carrier Transport Properties, and Self-Powered Photodetection. Adv. Opt. Mater. 2017, 5 (2), 1600704 10.1002/adom.201600704.

