# Supplementary material for: Spatial Localization of Defects in Halide Perovskites Using Photothermal Deflection Spectroscopy
Source: J Phys Chem Lett. 2024 Jan 26;15(5):1273–8. doi: 10.1021/acs.jpclett.3c02966 (PMC10860124; doi:10.1021/acs.jpclett.3c02966)
Supplement: Supplementary file 1 — jz3c02966_si_001.pdf [file jz3c02966_si_001.pdf]

Supporting Information: Spatial Localization of Defects in Halide Perovskites Using Photothermal Deflection Spectroscopy

Ales Vlk(1); Zdenek Remes(1); Lucie Landova(1); Katarina Ridzonova(1); Robert Hlavac(1); Antonin Fejfar (1); Martin Ledinsky(1)*

1. Institute of Physics of the Czech Academy of Sciences, Cukrovarnicka 10, 16200 Prague, Czech Republic.

AUTHOR INFORMATION

Corresponding Author

vlkal@fzu.cz

KEYWORDS bulk defects, surface defects, photothermal deflection spectroscopy, Urbach energy

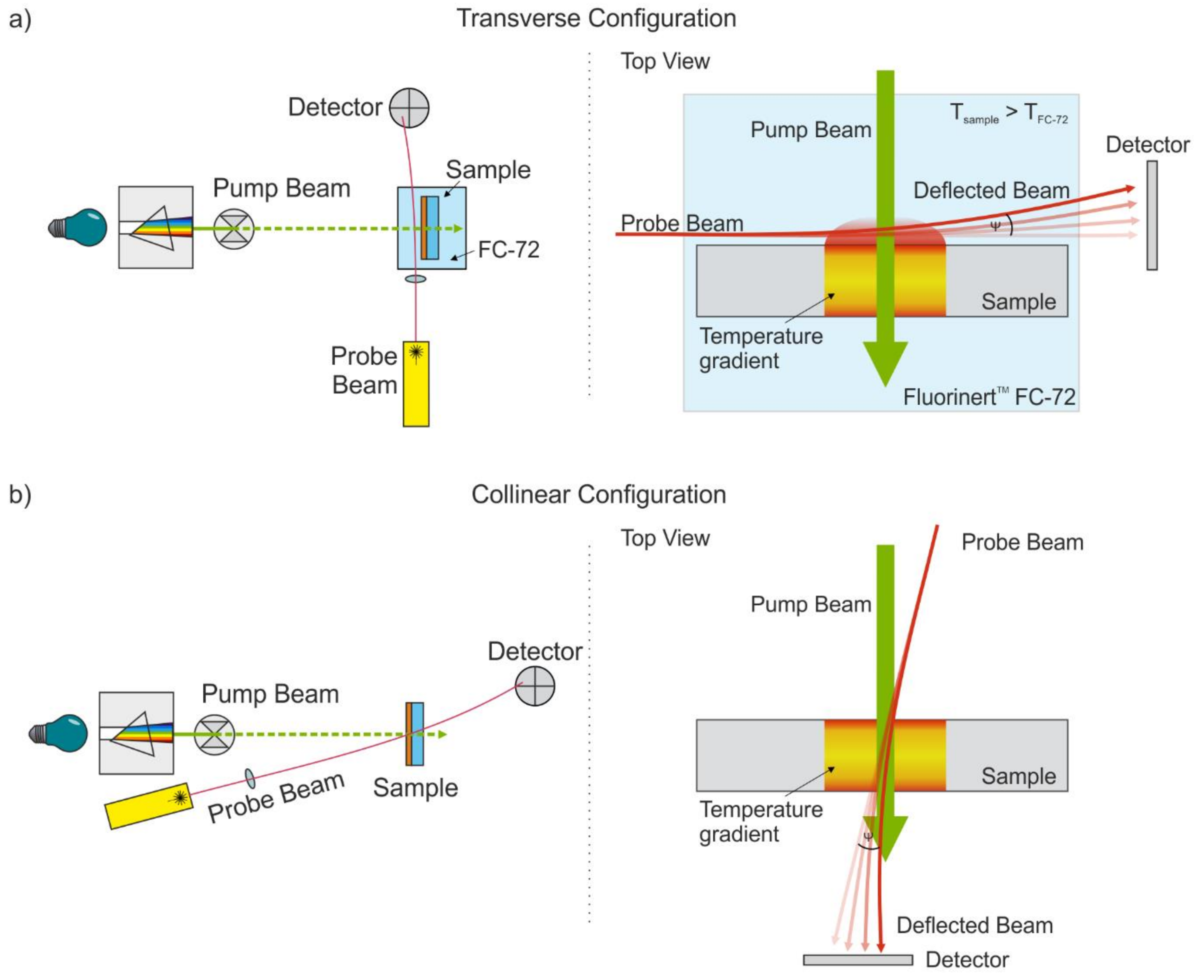

Figure S1. Schema of the two configurations of PDS setup: collinear a) and transverse b) including detailed view of the beam deflection inside/outside the sample.

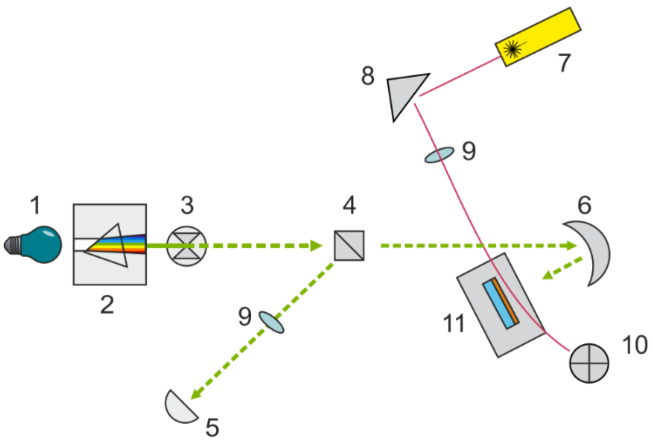

Figure S2. Schematic view of the custom-made PDS setup in transverse configuration at the Institute of Physics of the ASCR, v.v.i.: 1-light source with focusing optics, 2-monochromator and optical filters, 3-mechanical chopper, 4-beamspliter, 5-Si + InGaAs detector, 6-spherical mirror, 7-HeNe probe laser, 8-flat mirror, 9-focusing lenses, 10-position detector, 11-sample immersed in liquid, 12-anti-vibration table.

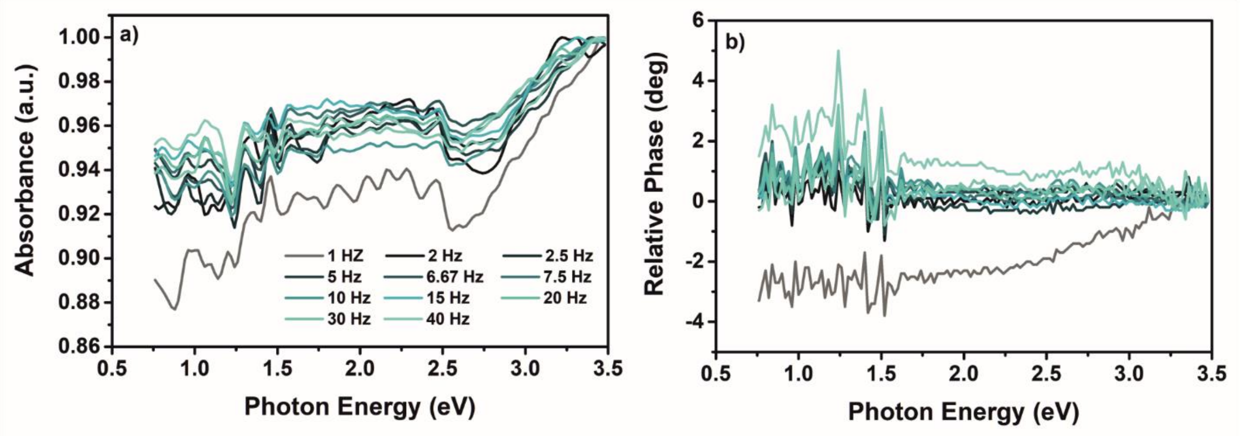

Figure S3. Absorption spectra a) and relative phase b) of carbon nanotubes reference sample measured at different chopping frequencies.

Preparation of $MAPbBr_3$ thin films

$MAPbBr_3$ thin film was deposited on a quartz substrate by spin-coating in a nitrogen glovebox. First, methylammonium bromide (MABr, 1 $mol.l^{-1}$) and lead bromide ($PbBr_2$, 1 $mol.l^{-1}$) were dissolved in a mixture of γ-butyrolactone (GBL) and dimethyl sulfoxide (DMSO) (3:2, v/v) by stirring overnight at 70 °C. Next, the filtrated hot solution (130 µl) was spin-coated onto the quartz substrate (20 mm x 20 mm) at 1,000 rpm for 12 s, and then the speed was gradually increased to 5,000 rpm and maintained for 40 s. After 30 s rotation, chlorobenzene (63 µl) was dropped on the film. Finally, the sample was annealed at 80 °C for 10 minutes.[1]

Preparation of $MAPbBr_3$ and Bi doped $MAPbBr_3$ crystals

$MAPbBr_3$ and Bi-doped $MAPbBr_3$ crystals were grown using inverse temperature crystallization in a nitrogen glovebox. At first, two precursor solutions (1 $mol.l^{-1}$) were prepared from the MABr and $PbBr_2$, or MABr and bismuth bromide ($BiBr_3$), in DMF, respectively, and heated overnight at 50 °C. Filtrated precursor solutions were mixed to obtain crystallization solutions with 0 and 4 mol% Bi content. Crystallization was done at temperatures between 78 and 92 °C.[2]

Preparation of $MAPbCl_3$ crystals

$MAPbCl_3$ crystals were grown using inverse temperature crystallization in a nitrogen glovebox. Briefly, methylammonium chloride (MACl, 1 mol.l-1) and lead chloride (PbCl2, 1 mol.l-1) were dissolved in a mixture of DMF and DMSO (1:1, v/v) at room temperature. The slightly turbid solution was filtered. The crystallization was performed at temperatures between 45 and 72 °C.[3]

Preparation of $CsPbBr_3$ crystals

$CsPbBr_3$ crystals were grown using inverse temperature crystallization in a nitrogen glovebox. First, a crystallization solution was prepared from cesium bromide (CsBr, 1 $mol.l^{-1}$) and $PbBr_2$ (2 $mol.l^{-1}$) in DMSO. The solution was stirred for 2 days at room temperature and then heated from

60 °C to 100 °C. The temperature was increased by 5 °C every 20 minutes (at 60 °C after 10 min). After 30 minutes of heating at 100 °C, the hot solution was filtrated. The crystallization was carried on at temperatures between 100 and 123 °C. Isolated crystals were washed with hot DMSO.[4]

Thin film frequency dependence

For the thin film, of a thickness D in the order of few hundreds of nanometers (200 - 300 nm) the thermal diffusion length μ_t is more than thousand times larger than its thickness for all used chopping frequencies ω, see **Tab. S2**. Therefore, the change of the frequency does not affect the measured absorptance spectra. In the range from 0.8 to 3.5 eV, the absorption coefficient of perovskites reaches the maximum value of approximately 10^5 cm^{-1} which corresponds to the absorption length $l_\alpha \approx 100$ nm.[5] Therefore, the measured signal originates from a major part of the sample for both, weak and strong, absorptions, respectively.

Sample	ρ [$kg \cdot m^{-3}$]	k [$W \cdot m^{-1} \cdot K^{-1}$]	C [$J \cdot kg^{-1} \cdot K^{-1}$]	Ref	μ_t^{10Hz} [μm]
SC-MAPbBr3	3834	0.37 – 0.51	378	[6–8]	246
TF-MAPbBr3	3834	0.39 ± 0.05	NA	[6,7]	NA
SC-MAPbI3	4119	0.34 ± 0.08	311	[6,7]	230
SC-MAPbCl3	3171	0.50 ± 0.12	511	[6,7]	248
SC-CsPbBr3	4834	0.46 ± 0.12	220	[6,7]	294
SC-FAPbBr3	3807	0.49 ± 0.12	444	[6,7]	240

Table S1. Density ρ, thermal conductivity k and specific heat C of selected perovskite materials obtained from the stated references and calculated thermal diffusion lengths for ω = 10 Hz.

ω [Hz]	1	2	5	7.5	10	15	20	30	40
$\mu_t^{MAPbBr_3}$ [μm]	779	551	348	284	246	201	174	142	123
$\mu_t^{TF\text{-}MAPbBr_3}$ [μm]	734	518	328	267	232	189	164	134	116

Table S2. Thermal diffusion lengths μ_t calculated from the thermal parameters from Tab. 1(k = 0.44 $W \cdot m^{-1} \cdot K^{-1}$) for different chopping frequencies ω via Eq.1.

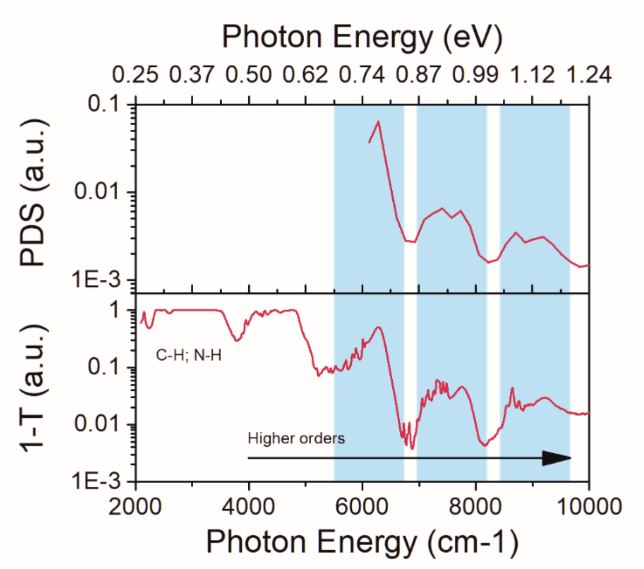

Figure S4. Comparison of FTIR-transmission and absorption spectra of $MAPbBr_3$ single crystal.

Calculation of Surface Temperature

To calculate the evolution of the surface temperature during the PDS measurement, we used 1D finite differences method in forward Euler scheme. Using this method, we solved following equation for change of the temperature in the volume i:

$$\frac{dT_i}{dt} = \alpha\left(-\frac{(T_i - T_{i-1})}{\Delta x^2} + \frac{T_{i+1} - T_i}{\Delta x^2}\right) + \frac{\alpha P}{\rho c}\exp(-\alpha x_i)\frac{1}{2}(\cos(2\pi\omega t) + 1), \quad \text{(S1)}$$

where T_i is a temperature in the volume i, α the absorption coefficient, $\Delta x = \frac{D}{n-1}$ is size of the finite difference element, i.e., sample thickness D over number of nodes n-1, P is the power of the

excitation source, ρ density of the sample, c specific heat, ω modulation frequency. The second term, $\frac{\alpha P}{\rho c}\exp(-\alpha x_i)\frac{1}{2}(\cos(2\pi\omega t)+1)$, of this equation represents heat deposited by absorption of mechanically chopped light.

The boundary conditions for heat transfer at the samples surface have following shape:

$$\frac{dT_{1/n}}{dt} = 2\alpha\frac{(T_{2/n-1}-T_{1/n})}{\Delta x^2} + \frac{2h}{\rho c\Delta x}(T_\infty - T_{1/n}) + \frac{\alpha P}{\rho c}\exp\left(-\alpha x_{1/n}\right)\frac{1}{2}(\cos(2\pi\omega t)+1), \quad \text{(S2)}$$

where h is a heat transfer coefficient. The lower indexes represent version of the condition for front (index 1) and back (index n) surface. In the **Fig. S4**, we can see the time dependent change of the surface temperature calculated using the previous equations for the set of parameters shown in **Tab. S3**.

D [μm]	n	T_0 [K]	T_∞ [K]	c [$J\cdot kg^{-1}\cdot K^{-1}$]
2050	510	300	300	378
ρ [$kg\cdot m^{-3}$]	ω [Hz]	h [$W\cdot m^{-2}\cdot K^{-1}$]	P [W/m^2]	k [$W\cdot m^{-1}\cdot K^{-1}$]
3834	5;10;40	500	10	0.51

Table S3. Parameters used in the calculation of the surface temperature.

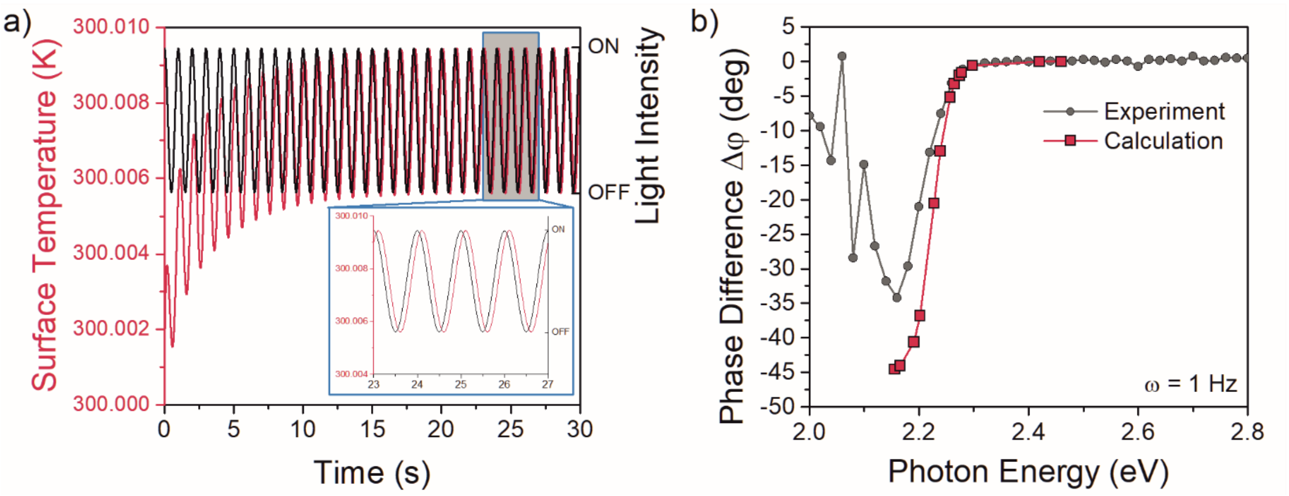

Figure S5. Comparison of time evolution of calculated surface temperature with the excitation light intensity a). The inset shows the delay of the surface temperature behind the excitation. Calculated and measured phase difference Δφ of the PDS signal for 1 Hz modulation frequency b).

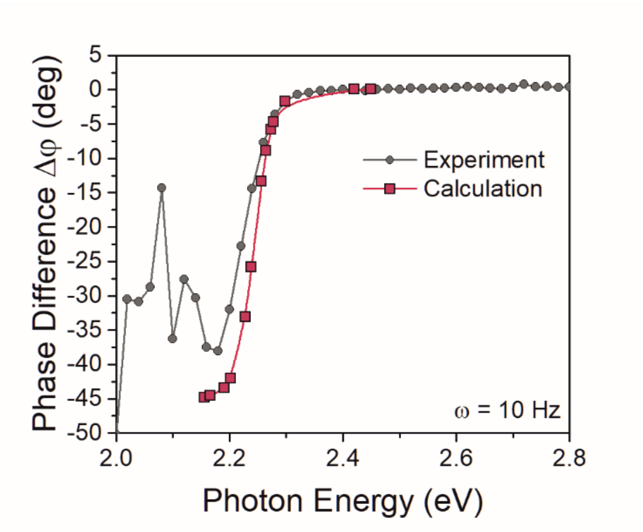

Figure S6. Calculated and measured phase difference Δφ of the PDS signal for 10 Hz modulation frequency.

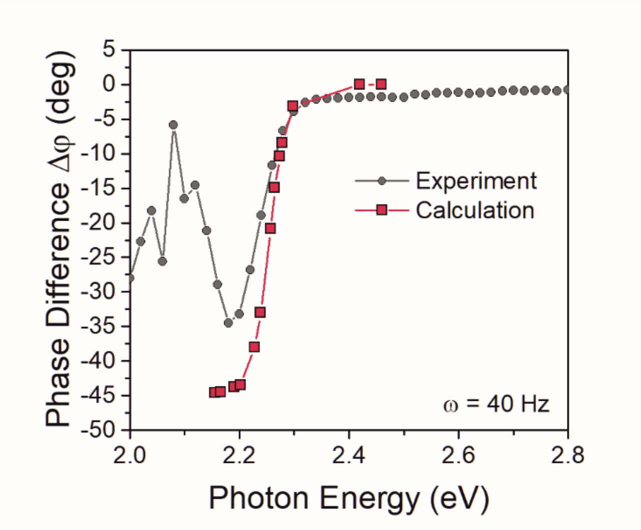

Figure S7. Calculated and measured phase difference Δφ of the PDS signal for 40 Hz modulation frequency.

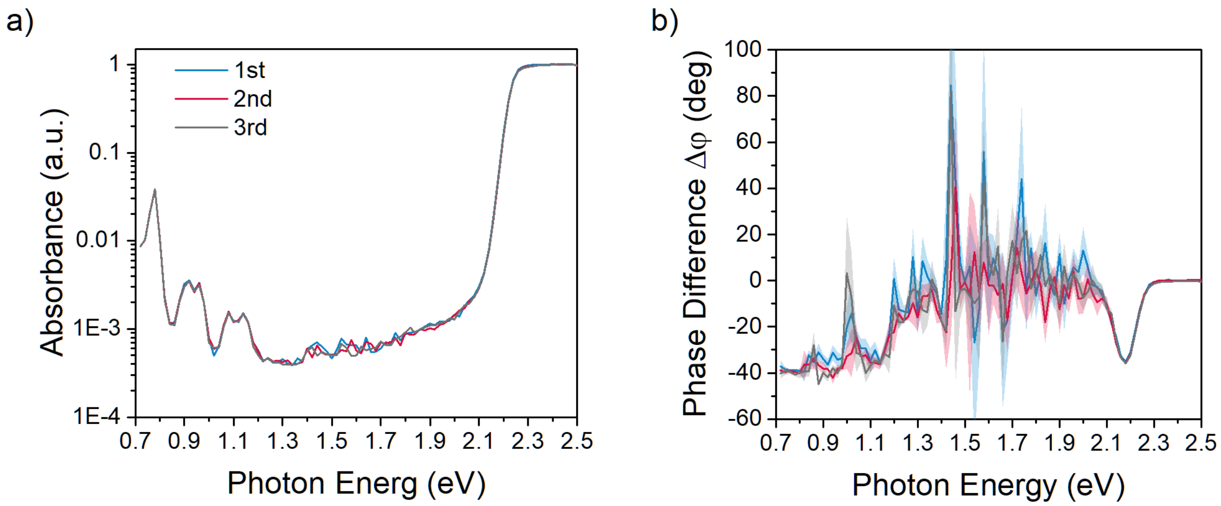

Figure S8. Comparison of three PDS spectra a) and phase difference b) of $MAPbBr_3$ single crystal measured at ω = 10 Hz after moving sample out of the optimal measurement position and repeating the alignment procedure between each measurement.

REFERENCES

(1) Holovský, J.; Peter Amalathas, A.; Landová, L.; Dzurňák, B.; Conrad, B.; Ledinský, M.; Hájková, Z.; Pop-Georgievski, O.; Svoboda, J.; Yang, T. C.-J.; Jeangros, Q. Lead Halide Residue as a Source of Light-Induced Reversible Defects in Hybrid Perovskite Layers and Solar Cells. *ACS Energy Lett.* **2019**, *4* (12), 3011–3017. https://doi.org/10.1021/acsenergylett.9b02080.

(2) Abdelhady, A. L.; Saidaminov, M. I.; Murali, B.; Adinolfi, V.; Voznyy, O.; Katsiev, K.; Alarousu, E.; Comin, R.; Dursun, I.; Sinatra, L.; Sargent, E. H.; Mohammed, O. F.; Bakr, O. M. Heterovalent Dopant Incorporation for Bandgap and Type Engineering of Perovskite Crystals. *J. Phys. Chem. Lett.* **2016**, *7* (2), 295–301. https://doi.org/10.1021/acs.jpclett.5b02681.

(3) Maculan, G.; Sheikh, A. D.; Abdelhady, A. L.; Saidaminov, M. I.; Haque, M. A.; Murali, B.; Alarousu, E.; Mohammed, O. F.; Wu, T.; Bakr, O. M. $CH_3NH_3PbCl_3$ Single Crystals: Inverse Temperature Crystallization and Visible-Blind UV-Photodetector. *J. Phys. Chem. Lett.* **2015**, *6* (19), 3781–3786. https://doi.org/10.1021/acs.jpclett.5b01666.

(4) Saidaminov, M. I.; Haque, M. A.; Almutlaq, J.; Sarmah, S.; Miao, X.-H.; Begum, R.; Zhumekenov, A. A.; Dursun, I.; Cho, N.; Murali, B.; Mohammed, O. F.; Wu, T.; Bakr, O. M. Inorganic Lead Halide Perovskite Single Crystals: Phase-Selective Low-Temperature Growth, Carrier Transport Properties, and Self-Powered Photodetection. *Adv. Opt. Mater.* **2017**, *5* (2), 1600704. https://doi.org/10.1002/adom.201600704.

(5) Leguy, A. M. A.; Azarhoosh, P.; Alonso, M. I.; Campoy-Quiles, M.; Weber, O. J.; Yao, J.; Bryant, D.; Weller, M. T.; Nelson, J.; Walsh, A.; van Schilfgaarde, M.; Barnes, P. R. F. Experimental and Theoretical Optical Properties of Methylammonium Lead Halide Perovskites. *Nanoscale* **2016**, *8* (12), 6317–6327. https://doi.org/10.1039/C5NR05435D.

(6) Elbaz, G. A.; Ong, W.-L.; Doud, E. A.; Kim, P.; Paley, D. W.; Roy, X.; Malen, J. A. Phonon Speed, Not Scattering, Differentiates Thermal Transport in Lead Halide Perovskites. *Nano Lett.* **2017**, *17* (9), 5734–5739. https://doi.org/10.1021/acs.nanolett.7b02696.

(7) Haeger, T.; Heiderhoff, R.; Riedl, T. Thermal Properties of Metal-Halide Perovskites. *J. Mater. Chem. C* **2020**, *8* (41), 14289–14311. https://doi.org/10.1039/D0TC03754K.

(8) Knop, O.; Wasylishen, R. E.; White, M. A.; Cameron, T. S.; Oort, M. J. M. V. Alkylammonium Lead Halides. Part 2. $CH_3NH_3PbX_3$ (X = Cl, Br, I) Perovskites: Cuboctahedral Halide Cages with Isotropic Cation Reorientation. *Can. J. Chem.* **1990**, *68* (3), 412–422. https://doi.org/10.1139/v90-063.
