# Supplementary material for: Spatial Localization of Defects in Halide Perovskites Using Photothermal Deflection Spectroscopy
Source: J Phys Chem Lett. 2024 Jan 26;15(5):1273–8. doi: 10.1021/acs.jpclett.3c02966 (PMC10860124; doi:10.1021/acs.jpclett.3c02966)
Supplement: Supplementary file 2 — jz3c02966_si_002.pdf [file jz3c02966_si_002.pdf]

jz-2023-029664.R1

Name: Peer Review Information for "Spatial Localization of Defects in Halide Perovskites Using Photothermal Deflection Spectroscopy"

First Round of Reviewer Comments

Reviewer: 1

Comments to the Author

Vlk et al. study organic and inorganic perovskite single crystals using PDS.

The presented study is highly interesting, yet a few things could improve the manuscript:

1. The authors first validate their system and look at the sub-bandgap signal of a $MAPbBr_3$ SC as a function of chopper frequency to assess the thermal diffusion length.

To this end, the authors subtract a "baseline signal" without explaining at all where this comes from, nor why the authors selected the provided lineshape, so where does this come from?

2. the authors could validate their approach by cross-checking the signal with back illumination (i.e. forcing thermal diffusion through the whole crystal (if it's not too thick)). There, especially the above-bandgap absorption would be important, of course. So a sort of thermal time-of-flight measurement, but instead looking at the frequency.

3. Next, the authors identify convincingly the origin of the sub-bandgap states by comparing different cation/halide systems. Again a question related to the background of the three different material systems: why does the background differ so much? Almost 2 orders of magnitude (Fig. 2)?

4. Next, the authors tend to study the phase behaviour of the signal as a function of the photon energy. This is rather elegant, but I am surprised there are no error bars on the measured data

5. In comparing the measured data to FEM simulation, the authors get reasonable agreement for photon energies close to or above bandgap, yet there is a systematic discrepancy further below.

So I'd rephrase the sentence: "Perfect agreement between the measured and calculated data validates our model description of the PDS experiment" which they later attempt to explain with "the incompleteness of our

numerical model". I think the authors are correct and with the Bi-doped samples, they provide a reasonable explanation for a "flat" phase difference as they see in Fig 4b, however, in the un-doped crystal, there appears to be another peak around 2.1 eV.

What happens for energies lower than that?

Also, did the authors consider that upon illuminating significantly below bandgap, they might create interference within the SC so that the signal stems from locally enhanced optical absorption in a comparably small fraction of the SC?

6. The authors finally look at degradation-induced changes in the PDS signal and the phase shift. I think their conclusions are sound, yet I'd suggest that they validate their conclusion that upon white light illumination, they create surface defects:

Compare SCs aged with blue light (forcing any degradation to be near the surface) with SCs aged with light illumination far in the bulk (so close to the band-edge). Alternatively, if the SCs are thin or can be made thin (e.g., cleaving with a knife works reasonably well, https://pubs.acs.org/doi/full/10.1021/acs.jpclett.8b03728), one could simply age (again with blue light) and then measure from the non-illuminated side, so essentially flip the SC.

typos/grammar:

"The electron hole can undergo"

electron-hole or electron hole

"thus creates a large amount of surface non-radiative recombination deep defects" is missing an adverb or sth

As written, overall very interesting manuscript. With a few additions, it's absolutely worth publishing.

Reviewer: 2

Comments to the Author

Manuscript by Vlk et al describes an application of the photothermal deflection spectroscopy (PTD) for detection of sub-bandgap states in single crystals of metal halide perovskites. The application of the method to perovskites looks quite interesting. Detection of these states is indeed important. The method is essentially a lock-in technique where the signal created by the deflection of the modulated beam is measured at the same frequency as its modulation. Using a peculiar dependence of the signal on the modulation frequency (due to the finite speed of heat propagation) the authors claim that they can distinguish light absorbing states close to the surface of the crystals from those in the bulk of the crystal. Potentially this method would be indeed very useful. Although the general ideal looks very appealing, the description of the method and of the obtained results leaves many questions open about the reliability of the data and their interpretation. Basically, before one can discuss the defect states in perovskites and their spatial location one need to prove that the method really works using a model sample with known properties. Major revision is needed to make this paper acceptable for publication in JPCLetters, see details below.

1) In general, the paper is written in such a style, that it is very difficult to understand all logic, all technical details and approximations. The text is rather long, but a large length does not mean a better description. Despite of the lengthy style and many figures, the manuscript gives an impression of a poorly presented work with many crucial details missing.

2) The proposed dependence on the modulation frequency needs to be described in detail. This is the key point of this work however it is not properly explained. Since the authors have a computer model, they could use it to explain the idea with the calculated dependencies including the heat distribution inside the material shown.

3) “PDS is a lock-in-based technique” is mentioned in page 12 of the manuscript. Without knowing this there cannot be any frequency dependence of the signal. The description of the methods is very poorly presented just because this key point is not presented in the very beginning.

4) Absorption spectra and spectra of PTD should be compared for samples, where the sub-bandgap absorption is measurable by a standard absorption spectrometer.

5) There are no errors bars in the spectra. How does a reader know that the oscillations (jumping) of the signal are not just due to noise or systematic errors?

6) Is there any refence sample to compare? Also, a sample where absorption at the surface and bulk is different (I can easily image how I would make such a sample using a colour glass filter with an extra light absorbing layer deposited to the surface).

7) Basically, before one can discuss the defect states in perovskites and their location one need to prove that the method really works using a model sample with known properties.

8) Fig,5. The features in the absorption spectra are so sharp, that it is extremely hard to imagine that they are real. How does the same spectrum look like for another crystal of the same type? How does it look like if the setup is mistuned and tuned again? How does the signal look like for as piece of glass? The same concerns all other figures.

9) There are no details about the setup like the size of the cell filled with liquid, power of the main light (which is absorbed by the sample), size of the sample, diameter of the beam and so on which is all necessary to be able to repeat the experiments.

10) The authors need to make a detailed picture (cartoon) of the processes of heating/absorption, heat diffusion, refractive index change (where ? in the liquid? In the crystal?) and the role of the modulation frequency. On one side, the authors obviously try to explain all as good as possible in long and often related passages (not fully successfully, but still) and at the same time they used the figure of the setup, which was designed not by them and, therefore, it does not explain what they what to explain.

11) How many crystals were measured? Several spectra of the same crystal measured at different alignments of the setup and spectra of different crystals of the same type should be shown.

12) Does the dependence on frequency make sense when the known heat conductivity of perovskite is considered?

13) There is no scale for the phase shift in Fig4 b.

14) How do I know that the waves what are seen in the spectra are not consequences of the interference of the light when it passes the crystals? Since the size of the crystals is not given, it is not possible to judge this problem.

15) Abstract: “vibrational and structural defect states” – what are these? Strange formulation.

16) Metal-halide perovskites are usually called MHP; abbreviation PK is strange.

17) Page 4. “In PKs thin films, the defect states optical emission activity as well as absorption-induced photoconductivity is minimal” This sentence does make much sense to me. What is “emission activity”? “Activity” is a strange term to apply to defects. Photoconductivity, by its definition, is always induced by absorption. What do the authors really mean by this sentence?

18) P.5 “Therefore, this method is highly sensitive to all non-radiative recombination absorption states”. What does “non-radiative recombination absorption states” mean? I have a hard time to imagine what it is. This type of language problem (unclear, confusing formulations) occurs in many places in this manuscript.

19) P.5 “transversal configuration of PDS”, what does “transversal” mean? All these terms are relative to something. Confusing.

20) The heat diffusion length (miu) is a confuting term. How can a diffusion length depend on the modulation frequency? The authors used a very general term (diffusion length) in the very specific case with the very specific restrictions, experimental conditions without explaining them. For example, the diffusion length of charge carriers does not depend on any modulation of excitation light intensity. Diffusion length depends on the diffusion coefficient and the lifetime of charge carriers. So, in the case of PTD spectroscopy, what is the “lifetime” of the “heatwave”? To my understanding, the dependence of “heat diffusion length” comes from the way the heat is detected. This is because PDF detects changes of temperature, not the increase of temperature as such.

Anyway, this part is very poorly explained, and it will be definitely impossible to understand for most of the readers.

21) The authors say that they can measure OD=10-4 (absorption). However, this is the usual limit of a good traditional absorption spectrometer.

22) It is not clear why low conductivity of the substrate is important.

23) p.9. Why is the heat going to propagate to the surface if, for example, the heat is uniformly generated through the whole thickness of the sample? Heat propagates anywhere is there is a gradient.

24) P.10, top. Why is there a difference between surface to bulk and bulk – to- surface heat transfer?

25) P.10. “As a result of the higher sensitivity of the measurement on the bulk MAPbBr3 SC in the low absorption region, we were able to observe two significant peaks at 0.925 and 1.110 eV”. I do not see any logic in this sentence.

26) p.12. The authors are talking about MA+. If one looks in the literature, these ions are the least people warry about. Defects related to MA seems to be not important for non-radiative recombination.

27) To my understanding, there is no direct connection between the absorption cross section of a defects and its role in charge recombination in a semiconductor.

28) Why the important defects (like iodide vacancies and interstitials) are not visible by PTD? Where are they?

29) The text is full of relative comparison like “a greater depth”, “closer to the surface”, how much greater? How much closer is it? What is the refence point, reference length?

30) What is the spatial resolution of the method (frequency dependence) in z-direction?

31) P.14. Why is the phase shift expected to be 45 degrees? Does it not depend on the thickness of the sample?

Reviewer: 3

Comments to the Author

This work illustrates how photothermal deflection spectroscopy (PDS) can be utilized to identify sub-band gap defects in the bulk and on the surface of the perovskites. The paper also elucidates the usefulness of the technique in identifying bulk sub-band gap defects arising due to Bi addition, while exposure to light led to the formation of surface defects on the perovskite single crystal. This study entailed the methodology for using PDS, especially for perovskites. However, there are some issues that are highlighted below and should be addressed. I believe incorporating these changes will further improve the quality of this manuscript. Therefore, I recommend publishing this paper after major revision.

1. Some details like single crystal thickness, absorption length, and thermal diffusion length for the different perovskite materials are missing. It should be tabulated in the main manuscript for the reader to understand the regime under which the measurements are performed.

2. All of the PDS measurements are done on high band gap perovskite materials with band gaps close to or greater than 2 eV, which are not usually utilized in state-of-the-art high-efficiency perovskite solar cells. Therefore, the viability of this technique should be checked on more widely studied and researched perovskites such as MAPbI3 and CsFAMa-based triple cation mixed halide perovskites that are used in high-efficiency perovskite solar cells.

3. In Figure 4 (b), a higher frequency is used to identify the surface defects within the band gap. But what are these surface defects? Please elaborate.

4. In Figure 5, delta phi is becoming greater than zero for a range of 1.2-2.0 eV. Please explain this. Is it normal to have delta phi greater than zero when surface defects are present?

5. Elaborate if this technique can be used to quantify the defects in bulk and on the surface of the perovskite.

6. The thickness range of the perovskite films that can be used for PDS measurement should be mentioned. All of the PDS measurements performed in this study are on thick single crystals. However, most perovskite solar cells are based on perovskite layers less than 1 micron in thickness. A preliminary analysis should be done using the absorption length, thermal diffusion coefficient, and film thickness to arrive at the range of thicknesses for which this technique can yield accurate results.

Author's Response to Peer Review Comments:

Dear editor and reviewers,

we are grateful for all the comments and suggestions, we did our best to answer and implement them. Please see the attachment for our full response.

Best regards,

Aleš Vlk and co-authors.

Reviewer(s)' Comments to Author:

Reviewer: 3

Recommendation: This paper may be publishable, but major revision is needed; I would like to be invited to review any future revision.

Comments:
This work illustrates how photothermal deflection spectroscopy (PDS) can be utilized to identify sub-band gap defects in the bulk and on the surface of the perovskites. The paper also elucidates the usefulness of the technique in identifying bulk sub-band gap defects arising due to Bi addition, while exposure to light led to the formation of surface defects on the perovskite single crystal. This study entailed the methodology for using PDS, especially for perovskites. However, there are some issues that are highlighted below and should be addressed. I believe incorporating these changes will further improve the quality of this manuscript. Therefore, I recommend publishing this paper after major revision.

Additional Questions:
Urgency: High

Significance: Top 10%

Novelty: Top 10%

Scholarly Presentation: High

Is the paper likely to interest a substantial number of physical chemists, not just specialists working in the authors' area of research?: Yes

---

Fist, we thank to the reviewer for his positive review. We did our best to answer all of his/her questions and address his/her suggestions in the manuscript.

1. Some details like single crystal thickness, absorption length, and thermal diffusion length for the different perovskite materials are missing. It should be tabulated in the main manuscript for the reader to understand the regime under which the measurements are performed.

Answer: Thank you for your suggestions. The Dimensions of the studied samples were added to the method section of the main manuscript, thermal diffusion lengths of other perovskite materials were added into the Tab. S1 in Supporting information.

2. All of the PDS measurements are done on high band gap perovskite materials with band gaps close to or greater than 2 eV, which are not usually utilized in state-of-the-art high-efficiency perovskite solar cells. Therefore, the viability of this technique should be checked on more widely studied and researched perovskites such as MAPbI3 and CsFAMa-based triple cation mixed halide perovskites that are used in high-efficiency perovskite solar cells.

Answer: Thank you for your suggestion. MAPbBr3 was chosen for the known Bi doping procedure as well as for the cubic shape of crystals and therefore easier mounting of the crystal into our sample holder. Moreover, this material is interesting for detector and other application as well. Additionally, the techniques introduced in this work are applicable on other perovskite materials (MAPbI3 and CsFAMa-based triple cation mixed halide perovskites) as well. We will target other materials in our future studies.

3. In Figure 4 (b), a higher frequency is used to identify the surface defects within the band gap. But what are these surface defects? Please elaborate.

Answer: Thank you for this question. It is known that possible surface defects for $MAPbI_3$ are iodine vacancies, or uncoordinated Pb and I atoms.(Lu, Haizhou, et al.) For $MAPbBr_3$ we expect similar defects. However, we are not able to determine which defects are causing the observed absorption.

Lu, Haizhou, et al. "Compositional and interface engineering of organic-inorganic lead halide perovskite solar cells." Iscience 23.8 (2020).

4. In Figure 5, delta phi is becoming greater than zero for a range of 1.2-2.0 eV. Please explain this. Is it normal to have delta phi greater than zero when surface defects are present?

Answer: Thank you for the comment. The phase difference is set to be zero for strong absorption. However, as the absorption depth is non-zero for the strong absorption, the phase difference may be greater than zero at the band gap, once the surface states are causing the absorption. The heat transfer is faster in such a case so there is even smaller phase difference between the pump beam and signal originating from the surface defects in comparison with strong absorption, which subsequently leads to positive phase difference values.

5. Elaborate if this technique can be used to quantify the defects in bulk and on the surface of the perovskite.
Answer: Thank you for the comment. Quantification of the defects is possible. The measured absorption itself is proportional to the number of absorption states. The nature of the defect states (bulk vs surface) can be distinguish using the phase difference analysis we present in this work. However, to determine the number/density of bulk defect states from the measurement on the thick single crystal, it is necessary to determine the probed volume, and precise calibration.

6. The thickness range of the perovskite films that can be used for PDS measurement should be mentioned. All of the PDS measurements performed in this study are on thick single crystals. However, most perovskite solar cells are based on perovskite layers less than 1 micron in thickness. A preliminary analysis should be done using the absorption length, thermal diffusion coefficient, and film thickness to arrive at the range of thicknesses for which this technique can yield accurate results.

Answer: Thank you for your comment. Comparison of the SC with standard thin film of a thickness of approximately 300 nm deposited on quartz substrate is shown in the Fig.1a. As explained in the manuscript, in the case of thin film, there is no dependency on the frequency as the μ_t is much larger than the thickness of the sample. However, there is no fundamental obstacle for use of the analysis of the behaviour of the phase difference in case of the standard perovskite thin films. Extension of this technique to thin film is beyond the scope of this manuscript. In the near future we are planning extensive study of the phase behaviour of perovskite thin films as well.

Reviewer: 4

Recommendation: This paper represents a significant new contribution and should be published as is.

Comments:
The manuscript by Vlk et al. reports the use of PDS to determine bulk and surface defects in perovskite crystals. This is a nice piece of work. I recommend its publication in JPCL.

Additional Questions:
Urgency: High

Significance: High

Novelty: High

Scholarly Presentation: High

Is the paper likely to interest a substantial number of physical chemists, not just specialists working in the authors' area of research?: Yes

---

We thank to the reviewer for his positive review.

Reviewer: 1

Recommendation: This paper may be publishable, but major revision is needed; I would like to be invited to review any future revision.

Comments:
Vlk et al. study organic and inorganic perovskite single crystals using PDS.
The presented study is highly interesting, yet a few things could improve the manuscript:

Additional Questions:

Urgency: High

Significance: Top 10%

Novelty: High

Scholarly Presentation: Top 10%

Is the paper likely to interest a substantial number of physical chemists, not just specialists working in the authors' area of research?: Yes

---

Fist, we thank to the reviewer for his positive review. We did our best to answer all of his/her questions and address his/her suggestions in the manuscript.

1. The authors first validate their system and look at the sub-bandgap signal of a MAPbBr3 SC as a function of chopper frequency to assess the thermal diffusion length.

To this end, the authors subtract a "baseline signal" without explaining at all where this comes from, nor why the authors selected the provided lineshape, so where does this come from?

Answer: Thank you for your suggestion. The sentence “In order to obtain the intensity, the baseline caused by free carrier absorption was removed and the peak was fitted using the Gaussian function.” was added into manuscript. The reason for gaussian line shape lies in its multi-phonon absorption origin, as discussed later.

2. the authors could validate their approach by cross-checking the signal with back illumination (i.e. forcing thermal diffusion through the whole crystal (if it's not too thick)). There, especially the above-bandgap absorption would be important, of course. So a sort of thermal time-of-flight measurement, but instead looking at the frequency.

Answer: Thank you for this suggestion, however as you expected, the crystal is too thick. The thickness of our samples is approximately 2 mm, which is roughly 10x more than the thermal diffusion length at 10 Hz.

3. Next, the authors identify convincingly the origin of the sub-bandgap states by comparing different cation/halide systems. Again a question related to the background of the three different material systems: why does the background differ so much? Almost 2 orders of magnitude (Fig. 2)?

Answer: The large difference between sub bandgap absorption of the $MAPbCl_3$ SC and the other two samples ($MAPbBr_3$ a $CsPbBr_3$) is caused due to lower quality of the Cl based SC. From the phase difference, see figure bellow, we can see that between 2.55 and 1.40 eV, the phase difference reaches $|\Delta\ \phi|$=0 deg. This suggests presence of high number of surface defects.

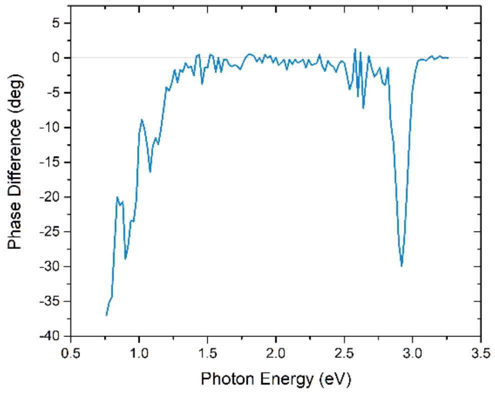

4. Next, the authors tend to study the phase behaviour of the signal as a function of the photon energy. This is rather elegant, but I am surprised there are no error bars on the measured data.

Answer: Thank you for your comment, we added the error bars for the phase measurements.

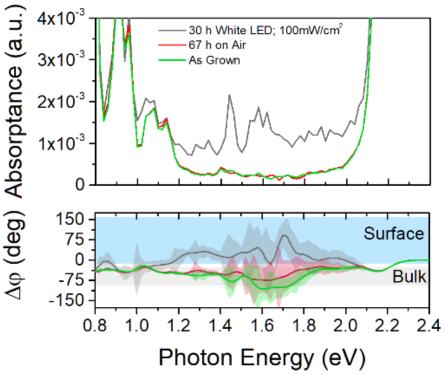

5. In comparing the measured data to FEM simulation, the authors get reasonable agreement for photon energies close to or above bandgap, yet there is a systematic discrepancy further below.

So I'd rephrase the sentence: "Perfect agreement between the measured and calculated data validates our model description of the PDS experiment" which they later attempt to explain with "the incompleteness of our numerical model". I think the authors are correct and with the Bi-doped samples, they provide a reasonable

explanation for a "flat" phase difference as they see in Fig 4b, however, in the un-doped crystal, there appears to be another peak around 2.1 eV. What happens for energies lower than that?

Also, did the authors consider that upon illuminating significantly below bandgap, they might create interference within the SC so that the signal stems from locally enhanced optical absorption in a comparably small fraction of the SC?

Answer: Thank you for the comment. The sentence "Perfect agreement between the measure" was rephrased to "Good agreement between the measured and calculated data in the strong absorption region and the absorption edge validates our model description of the PDS experiment." We believe that the interference does not play role in our measurement as the thickness of the crystal is around 2 mm. We added the dimensions of the samples in the method section of the main manuscript. From our observations we noticed, it is common that if there is no strong absorption in the bulk of the sample, the surface defects (which are always present in the un-pasivated surface) cause decrease of the phase shift, i.e. the peak at 2.1 eV in this case. For the energies bellow, a strong contribution of the phonon absorption (CH and NH vibrational modes in Fig. 2) causes the phase difference increase.

6. The authors finally look at degradation-induced changes in the PDS signal and the phase shift. I think their conclusions are sound, yet I'd suggest that they validate their conclusion that upon white light illumination, they create surface defects:

Compare SCs aged with blue light (forcing any degradation to be near the surface) with SCs aged with light illumination far in the bulk (so close to the band-edge). Alternatively, if the SCs are thin or can be made thin (e.g., cleaving with a knife works reasonably well, https://pubs.acs.org/doi/full/10.1021/acs.jpclett.8b03728), one could simply age (again with blue light) and then measure from the non-illuminated side, so essentially flip the SC.

Answer: The studied sample is approximately 2 mm thick, therefore, the absorbed light will not affect the non-illuminated side of the sample. Moreover, even for used white LED illumination, majority of the light is absorbed near the surface, thus the degradation starts from the surface.

Typos/grammar:

"The electron hole can undergo"

electron-hole or electron hole

"thus creates a large amount of surface non-radiative recombination deep defects" is missing an adverb or sth
Answer: Thank you for your corrections. We corrected the mentioned typos and grammar mistakes.

As written, overall very interesting manuscript. With a few additions, it's absolutely worth publishing.

Reviewer: 2

Recommendation: This paper may be publishable, but major revision is needed; I would like to be invited to review any future revision.

Comments:
Manuscript by Vlk et al describes an application of the photothermal deflection spectroscopy (PTD) for detection of sub-bandgap states in single crystals of metal halide perovskites. The application of the method to perovskites looks quite interesting. Detection of these states is indeed important. The method is essentially a lock-in technique where the signal created by the deflection of the modulated beam is measured at the same frequency as its modulation. Using a peculiar dependence of the signal on the modulation frequency (due to the finite speed

of heat propagation) the authors claim that they can distinguish light absorbing states close to the surface of the crystals from those in the bulk of the crystal. Potentially this method would be indeed very useful. Although the general ideal looks very appealing, the description of the method and of the obtained results leaves many questions open about the reliability of the data and their interpretation. Basically, before one can discuss the defect states in perovskites and their spatial location one need to prove that the method really works using a model sample with known properties. Major revision is needed to make this paper acceptable for publication in JPCLetters, see details below.

Additional Questions:

Urgency: Moderate

Significance: High

Novelty: Moderate

Scholarly Presentation: Moderate

Is the paper likely to interest a substantial number of physical chemists, not just specialists working in the authors' area of research?: Yes

---

Fist, we thank to the reviewer for his positive review. We did our best to answer all of his/her questions and address his/her suggestions in the manuscript.

1) In general, the paper is written in such a style, that it is very difficult to understand all logic, all technical details and approximations. The text is rather long, but a large length does not mean a better description. Despite of the lengthy style and many figures, the manuscript gives an impression of a poorly presented work with many crucial details missing.

Answer: Thank you for this comment. We tried to improve the text of the main manuscript.

2) The proposed dependence on the modulation frequency needs to be described in detail. This is the key point of this work however it is not properly explained. Since the authors have a computer model, they could use it to explain the idea with the calculated dependencies including the heat distribution inside the material shown.

Answer: Thank you for this comment. The dependence of the thermal diffusion length on the modulation frequency comes from its definition and it is described in detail in reference 14.

[14] Rosencwaig, A.; Gersho, A. Theory of the Photoacoustic Effect with Solids. *J. Appl. Phys.* 1976, *47* (1), 64–69. https://doi.org/10.1063/1.322296.

3) "PDS is a lock-in-based technique" is mentioned in page 12 of the manuscript. Without knowing this there cannot be any frequency dependence of the signal. The description of the methods is very poorly presented just because this key point is not presented in the very beginning.

Answer: Thank you for the comment. We added the fact, that the PDS is a lock-in-base technique into the introduction of the article. Moreover, we modified the introduction and description of the method and supported the explanation with figure in SI.

4) Absorption spectra and spectra of PTD should be compared for samples, where the sub-bandgap absorption is measurable by a standard absorption spectrometer.

Answer: Thank you for your comment. PDS itself is relatively common technique. We kindly refer you to our other work, where we compare several techniques for absorption measurements, E. Ugur et al.

Ugur, E.; Ledinský, M.; Allen, T. G.; Holovský, J.; Vlk, A.; De Wolf, S. Life on the Urbach Edge. *J. Phys. Chem. Lett.* 2022, *13* (33), 7702–7711. https://doi.org/10.1021/acs.jpclett.2c01812.

5) There are no errors bars in the spectra. How does a reader know that the oscillations (jumping) of the signal are not just due to noise or systematic errors?

Answer: Thank you for your comment, we added the error bars for the phase measurements.

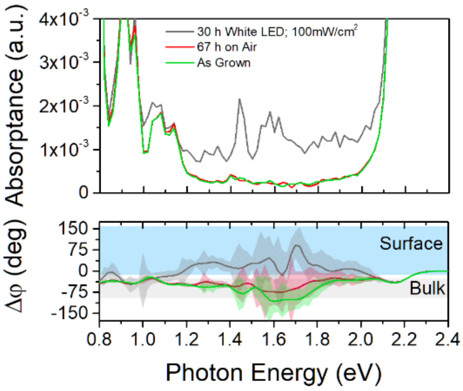

6) Is there any refence sample to compare? Also, a sample where absorption at the surface and bulk is different (I can easily image how I would make such a sample using a colour glass filter with an extra light absorbing layer deposited to the surface).

Answer: Thank you for the interesting idea. We did not perform such experiment. We believe that our examples of bismuth doping (bulk defects) and surface degradation prove together with presented calculations that suggested method works.

7) Basically, before one can discuss the defect states in perovskites and their location one need to prove that the method really works using a model sample with known properties.

Answer: Thank you for your comment. This comment was answered together with previous question. We have specifically chosen MAPbBr3 as a model material due to our extensive experience with this material.

8) Fig,5. The features in the absorption spectra are so sharp, that it is extremely hard to imagine that they are real. How does the same spectrum look like for another crystal of the same type? How does it look like if the setup is mistuned and tuned again? How does the signal look like for as piece of glass? The same concerns all other figures.

Answer: Thank you for your comment. The resolution in energy is 0.02 eV, therefore, those features may appear sharper than they really are. For example, for the peak at 1.44 eV, there is 5 points. To prevent random spikes appearing in the PDS spectra, each point of the spectra is acquired several times, and a median is taken. The PDS spectra and phase dependence of calibration sample are shown in the SI.

9) There are no details about the setup like the size of the cell filled with liquid, power of the main light (which is absorbed by the sample), size of the sample, diameter of the beam and so on which is all necessary to be able to repeat the experiments.

Answer: Thank you for your comment. Thank you for the comment, we added those parameters into the methods section of the manuscript.

10) The authors need to make a detailed picture (cartoon) of the processes of heating/absorption, heat diffusion, refractive index change (where ? in the liquid? In the crystal?) and the role of the modulation frequency.

On one side, the authors obviously try to explain all as good as possible in long and often related passages (not fully successfully, but still) and at the same time they used the figure of the setup, which was designed not by them and, therefore, it does not explain what they what to explain.

Answer: Thank you for your suggestion, we provided a modified picture in the SI.

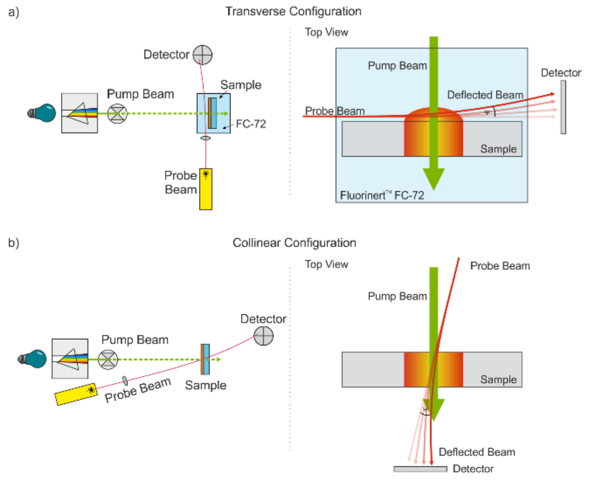

11) How many crystals were measured? Several spectra of the same crystal measured at different alignments of the setup and spectra of different crystals of the same type should be shown.

Answer: Thank you for your comment: The measurement is repeatable. We have performed the same measurement on different samples and the results are in good agreement. It is not possible to repeat the measurement with different alignments as the measurement is done on optimized and calibrated setup.

12) Does the dependence on frequency make sense when the known heat conductivity of perovskite is considered?

Answer: Thank you for your question. The presented frequency dependence of the thermal diffusion length is very well in line with the theoretical prediction from the REF. 14 (Eq. 4).

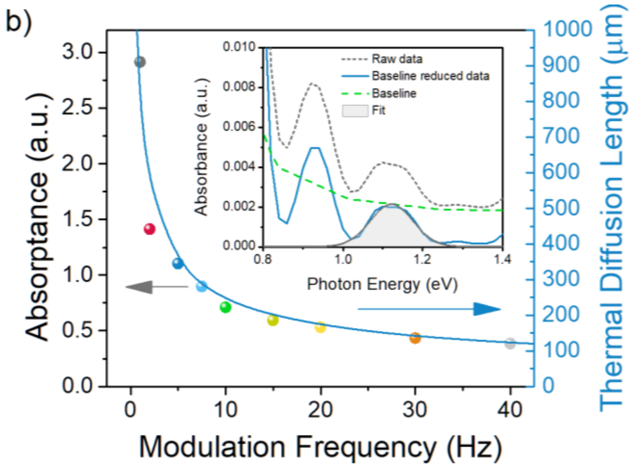

13) There is no scale for the phase shift in Fig4 b.

Answer: Thank you for the comment, the scale was added to the Fig. 4b).

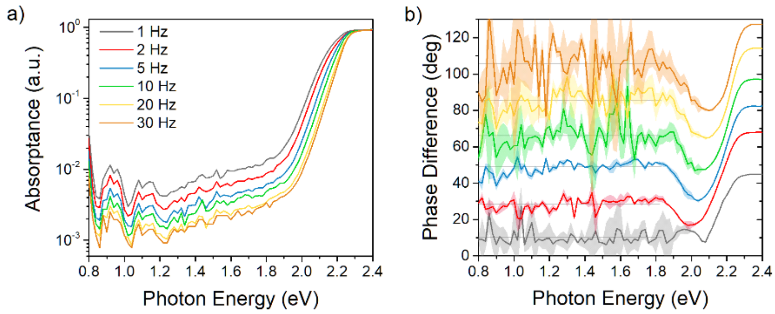

14) How do I know that the waves what are seen in the spectra are not consequences of the interference of the light when it passes the crystals? Since the size of the crystals is not given, it is not possible to judge this problem.

Answer: Thank you for your comment. We added the dimensions of our samples in the method section of the main manuscript. The thickness of the crystals used in our measurements is approximately 2 mm. Therefore, the effects we observe and discuss are not consequence of an interference.

15) Abstract: “vibrational and structural defect states” – what are these? Strange formulation.

Answer: Thank you for your comment. The sentence was rephrased "The identified absorption states are attributed to vibrational states and structural defects, and their influence on the non-radiative recombination probability is discussed. "

16) Metal-halide perovskites are usually called MHP; abbreviation PK is strange.

Answer: Thank you for your comment, however, there is no consensus how to call the group of methyl ammonium lead halide perovskites. We can find in the literature different names: lead-halide perovskites (LHP), metal-halide perovskites (MHP), organic-inorganic halide perovskites (OHP) and many more. Therefore, we decided to use simple and short abbreviation PKs referring to APbX3 perovskites (where A = MA, Cs; X = Br, Cl in our case).

17) Page 4. “In PKs thin films, the defect states optical emission activity as well as absorption-induced photoconductivity is minimal” This sentence does make much sense to me. What is “emission activity”? “Activity” is a strange term to apply to defects. Photoconductivity, by its definition, is always induced by absorption. What do the authors really mean by this sentence?

Answer: Thank you for your comment. The sentence was rephrased "In PKs thin films, the defect states radiative emission activity, as well as absorption-induced photoconductivity, are low."

18) P.5 “Therefore, this method is highly sensitive to all non-radiative recombination absorption states”. What does “non-radiative recombination absorption states” mean? I have a hard time to imagine what it is. This type of language problem (unclear, confusing formulations) occurs in many places in this manuscript.

Answer: Thank you for the comment. We agree that the formulation is confusing. Therefore, we rewrote the sentence to “Therefore, this method is highly sensitive to all absorption states which serves as non-radiative recombination centres.”.

19) P.5 “transversal configuration of PDS”, what does “transversal” mean? All these terms are relative to something. Confusing.

Answer: Thank you for the comment. We modified the manuscript accordingly. The two possible geometries of PDS measurement were explained in the introduction and figure was added to SI to graphically support our explanation.

20) The heat diffusion length (miu) is a confuting term. How can a diffusion length depend on the modulation frequency? The authors used a very general term (diffusion length) in the very specific case with the very specific restrictions, experimental conditions without explaining them. For example, the diffusion length of charge carriers does not depend on any modulation of excitation light intensity. Diffusion length depends on the diffusion coefficient and the lifetime of charge carriers. So, in the case of PTD spectroscopy, what is the “lifetime” of the “heatwave”? To my understanding, the dependence of “heat diffusion length” comes from the way the heat is detected. This is because PDF detects changes of temperature, not the increase of temperature as such. Anyway, this part is very poorly explained, and it will be definitely impossible to understand for most of the readers.

Answer: Thermal diffusion length is frequently used term in the thermal wave propagation theory and field of photothermally based spectroscopies. We slightly reformulated our explanation. Originally: "The thermal diffusion length μt defines the characteristic length scale of heat transfer in the material and is affected by several parameters: pump beam modulation (chopping) frequency ω [Hz], sample density ρ [kg·m−3], specific heat capacity C [J·kg−1·K−1] and thermal conductivity k [W·m−1·K−1], see Eq. 1.8". Now: "The thermal diffusion length μt defines the characteristic length scale (distance over which) the heat change in the material propagates. It is inversely proportional to the thermal diffusion coefficient, and it depends on the pump beam modulation (chopping) frequency ω [Hz], see Eq. 1.14 Larger value of μ_t means that the heat can propagate to the detection point from greater distance."

21) The authors say that they can measure OD=10-4 (absorption). However, this is the usual limit of a good traditional absorption spectrometer.

Answer: Thank you for your comment. In the manuscript we state the dynamic range of PDS is 4 orders of absorption. However, we do not claim that it is significantly better than other methods. The advantage of the method in comparison with others is that it is sensitive to all absorption states. In PDS, no electron/hole pair collection (in case of FTPS) or radiative recombination (PL spectroscopy) is needed. Nevertheless, in comparison with transmission/reflection measurements, the sensitivity of PDS is much better (Ugur, et al.) especially in case of thick samples as ours.

Ugur, E.; Ledinský, M.; Allen, T. G.; Holovský, J.; Vlk, A.; De Wolf, S. Life on the Urbach Edge. *J. Phys. Chem. Lett.* 2022, *13* (33), 7702–7711. https://doi.org/10.1021/acs.jpclett.2c01812.

22) It is not clear why low conductivity of the substrate is important.

Answer: Thank you for the comment. The low thermal conductivity of substrate prevents dissipation of the heat into the substrate and thus increases the sensitivity of the measurement.

23) p.9. Why is the heat going to propagate to the surface if, for example, the heat is uniformly generated through the whole thickness of the sample? Heat propagates anywhere is there is a gradient.

Answer: Thank you for your comment. As the sample is immersed in the liquid FC67, the heat will propagate from the sample to the colder liquid. This causes a large temperature gradient at the surface of the PK SC and thus the heat will propagate from the bulk towards the surface.

24) P.10, top. Why is there a difference between surface to bulk and bulk – to- surface heat transfer?

Answer: Thank you for your question. The heat generated at the surface is transferred directly into the liquid. On the other hand, the heat generated in the bulk of the sample has to propagate towards the surface and then transfer into the liquid. This causes a time delay and thus phase shift between the signal generated at the surface and in the bulk of the sample.

25) P.10. “As a result of the higher sensitivity of the measurement on the bulk MAPbBr3 SC in the low absorption region, we were able to observe two significant peaks at 0.925 and 1.110 eV”. I do not see any logic in this sentence.

Answer: Thank you for your comment. We rewrote the sentence as "As a result of better signal-to-noise ratio of the measurement on the bulk MAPbBr3 SC in comparison with thin film, we were able to observe two significant peaks at 0.925 and 1.110 eV."

26) p.12. The authors are talking about MA+. If one looks in the literature, these ions are the least people warry about. Defects related to MA seems to be not important for non-radiative recombination.

Answer: Thank you for your comment. This fact is stated in the manuscript: "The MA+ related absorption states around the middle of the MAPbBr3 bandgap are potentially non-radiative recombination centres for charge carriers. But since these are multi-phonon absorption states, the probability of the non-radiative recombination is very low, as described by Kirchartz et al.20 Therefore, the MA+ related states do not significantly affect the transport properties and the open circuit voltage of the finalized solar cell."

27) To my understanding, there is no direct connection between the absorption cross section of a defects and its role in charge recombination in a semiconductor.

Answer: Thank you for your comment. We agree with your statement. We are not aware that opposite statement is presented in our manuscript. However, it is important to document and study all defect states.

28) Why the important defects (like iodide vacancies and interstitials) are not visible by PTD? Where are they?

Answer: Thank you for the question. The Br vacancies form very shallow defects close to the conduction band. Therefore, those states are hidden in the states at the absorption edge, see:

MOTTI, Silvia G., et al. Defect activity in lead halide perovskites. Advanced Materials, 2019, 31.47: 1901183.

29) The text is full of relative comparison like "a greater depth", "closer to the surface", how much greater? How much closer is it? What is the refence point, reference length?

Answer: Thank you for the comment. We believe that those relative comparisons are used only when such a general comment is helpful to understand the discussed problematics. We minimized the number of these relative comparisons in the text.

30) What is the spatial resolution of the method (frequency dependence) in z-direction?

Answer: Values of thermal diffusion length in MAPbBr3 for different frequencies are shown in the Fig. 1b and in the table Tab. S2 in SI. As the dependence of the thermal diffusion length is inversely proportional to the square root of the modulation frequency, the resolution (change of μ_t with omega) is not constant. The difference in mu at 10 Hz and 11 Hz is approximately 10 microns.

31) P.14. Why is the phase shift expected to be 45 degrees? Does it not depend on the thickness of the sample?

Answer: Thank you for this question. The thickness of our sample is approximately 2 mm. Therefore, the thickness dependence of the maximal phase shift considered as the sample is much thicker than the thermal diffusion length in this material. However, in general this phase shift varies with the thermal conductivity for different materials.

jz-2023-029664.R2

Name: Peer Review Information for "Spatial Localization of Defects in Halide Perovskites Using Photothermal Deflection Spectroscopy"

Second Round of Reviewer Comments

Reviewer: 3

Comments to the Author

All of the suggestions have been incorporated and reviewer comments have been addressed in the manuscript for the fulfilment of requisite quality of the journal. Hence, I recommend the publication of this work.

Reviewer: 2

Comments to the Author

The authors did rather mild changes in the manuscript taking into account the number of questions asked. When a reviewer asks a question, it means that some changes (explanations) should be added to the text. In rare cases, when the answer to the question is obvious for most of the readers, just an answer to the reviewer is enough. I do not think that in the case of this particular manuscript the latter case is applicable. So, I am not happy with the authors approach to the revision. Still, my concern about the accuracy of the measurements and possible random and systematic errors remains due to absence of any reference sample. The authors need to address it. Note, metal-halide perovskites cannot be references due to their instability. See more details below.

1) Page 8 “In order to obtain the intensity, the baseline caused by free carrier absorption was removed…” Why are the authors sure about the origin (free-carrier absorption) of this base line? Any measurement has an offset due to its technical realization. This base line may have nothing to do with the sample itself. As I have already mentioned, the problem of this study is that the authors do not show any refence measurements and do not discuss errors and limitations of the technique.

2) Fig.1 a. The authors assigned peaks at 0.9 and 1,1 eV to real transitions. What is about the peak at 1.45 eV? Is it real or not? It is visible at most of the curves, but not at all of them. It is visible at the

lowest and the highest frequency, but not at the middle ones. So, one needs to conclude that this peak is an artefact. In this case, why the other two peaks (0.9 and 1.1 eV) are not artefacts? All these peaks (0.9, 1.1 and 1.45) have about the same amplitude. There are many more “peaks” from 1.6 to 2 eV, are these peaks noise?

The authors must discuss this in details, and clearly motivate what they consider as real signal and what not and explain why.

3) Fig.1 b. The figure cation does not tell what is presented in the panel b. The text is confusing, it should be extended and re-phrased. Also, the panel a shows the single crystal and the film, what is the sample shown in b) ? How one can possibly understand that the authors plot in Fig.1b something related to the “fit” function which was calculated for all frequencies? This is just one example of the “rebuses” the authors give to the readers. To understand the basics of a figure one does not need to read the whole paper.

4) Abstract “The identified absorption states are attributed to vibrational states and structural defects, and their influence on the non-radiative recombination probability is discussed”

Vibrational states do not exist in vacuum. What are these states? States of what? Molecules? Ions?

5) “PKs thin films, the defect states radiative emission activity, as well as absorption-induced photoconductivity, are low.”

What does defect activity mean? This is slang, not a scientific language. Absorption induced photoconductivity ? Photoconductivity of MHP is very high as soon as you excite above the bandgap.

6) Question 4 from my previous report

Absorption spectra and spectra of PTD should be compared for samples, where the sub-bandgap absorption is measurable by a standard absorption spectrometer.

Answer: Thank you for your comment. PDS itself is relatively common technique. We kindly refer you to our other work, where we compare several techniques for absorption measurements, E. Ugur et al.

Ugur, E.; Ledinský, M.; Allen, T. G.; Holovský, J.; Vlk, A.; De Wolf, S. Life on the Urbach Edge. J. Phys. Chem. Lett. 2022, 13 (33), 7702–7711. https://doi.org/10.1021/acs.jpclett.2c01812”

Reviewer: I looked at the paper suggested and did not find the answer to my question. I am asking about the particular sample, not any other one reported in another papers. As the authors can easily read from my questions, I simply do not fully trust the results they try to sell, and this the problem of the authors to convince me. So, my request is very simple: absorption spectra of the

crystal measured using a traditional absorption spectrometer should be plotted together with the spectrum of the very same crystal obtained by PTD method.

7) Question 5 from my previous report

There are no errors bars in the spectra. How does a reader know that the oscillations (jumping) of the signal are not just due to noise or systematic errors?

Answer: Thank you for your comment, we added the error bars for the phase measurements.

Reviewer: How were these bars (I do not see any bars. I see some shadowed regions) were estimated? How many spectra were used to estimate this “bars”?

8) Fig. 4 b. The phase difference shows huge fluctuations over the spectrum. Are these fluctuations noise or not? Does only the offset (averaged over energy) have the meaning here? This must be mentioned in the fig caption.

9) Question 6 from my previous report

Is there any refence sample to compare? Also, a sample where absorption at the surface and bulk is different (I can easily image how I would make such a sample using a colour glass filter with an extra light absorbing layer deposited to the surface).

Answer: Thank you for the interesting idea. We did not perform such experiment. We believe that our examples of bismuth doping (bulk defects) and surface degradation prove together with presented calculations that suggested method works.

Reviewer: This is a bold statement which I totally disagree with. Surface degradation changes the morphology of the crystals and induce scattering. You cannot be sure that degradation occurs at the surface only. MHP cannot be used to test methods. This is because MHP are so strange materials, the materials which change their properties all the time. For example, why is the bismuth doping is a bulk doping? How can it be proved? It is just a hypothesis. The experiment I suggested is crucial (and easy) and I do not see why the authors cannot do it if they are sure about their technique.

10) Question 7 from my previous report

Basically, before one can discuss the defect states in perovskites and their location one need to prove that the method really works using a model sample with known properties.

Answer: Thank you for your comment. This comment was answered together with previous question. We have specifically chosen MAPbBr3 as a model material due to our extensive experience with this material.

Reviewer: I am dissatisfied with this answer. Perovskites are extremely difficult materials to work with. If the authors indeed have experience, as they claim, they know about it. I request the experiment with a dummy sample with different surface and bulk absorption (see above)

11) Question 8 from my previous report

Fig,5. The features in the absorption spectra are so sharp, that it is extremely hard to imagine that they are real. How does the same spectrum look like for another crystal of the same type? How does it look like if the setup is mistuned and tuned again? How does the signal look like for as piece of glass? The same concerns all other figures.

Answer: Thank you for your comment. The resolution in energy is 0.02 eV, therefore, those features may appear sharper than they really are. For example, for the peak at 1.44 eV, there is 5 points. To prevent random spikes appearing in the PDS spectra, each point of the spectra is acquired several times, and a median is taken. The PDS spectra and phase dependence of calibration sample are shown in the SI.

Reviewer: What are these “random spikes”? So, the method can give “random spikes”, is it so? Why the peaks I see in each spectrum are also not random spikes? Or systematic spikes? It all comes back to the errors and convincing the readers that the signals are real, and clearly marking the signals which are obvious artefacts.

11) Question 10 from my previous report

The authors need to make a detailed picture (cartoon) of the processes of heating/absorption, heat diffusion, refractive index change (where ? in the liquid? In the crystal?) and the role of the modulation frequency. On one side, the authors obviously try to explain all as good as possible in long and often related passages (not fully successfully, but still) and at the same time they used the figure of the setup, which was designed not by them and, therefore, it does not explain what they what to explain.

Answer: Thank you for your suggestion, we provided a modified picture in the SI

Reviewer: Details about the setup are given indeed. However, nothing understandable (orange-coloured regions?) is given about the heat diffusion. This needs to be done. I was asking about the physical picture which would also explain the diffusion length and the frequency effect which is central for this study.

12) Question 11 from my previous report

How many crystals were measured? Several spectra of the same crystal measured at different alignments of the setup and spectra of different crystals of the same type should be shown.

Answer: Thank you for your comment: The measurement is repeatable. We have performed the same measurement on different samples and the results are in good agreement. It is not possible to repeat the measurement with different alignments as the measurement is done on optimized and calibrated setup.

Reviewer: First of all, I would like to see experiments measured on different samples (especially crystals).

Second, of course, it is possible to measure at not identical conditions of the setup. This is how random errors originated form the setup alignment are estimated. It is very simple. You come to the lab next day. Tune the setup again - move all possible knobs out of the optimum and optimize the signal again. Place the sample in (the sample should be taken out and then set in again). All these manipulations are needed for any experimental technique to ensure that two measurements can be considered independent (or as independent as possible).

13) Question 16 from my previous report

Metal-halide perovskites are usually called MHP; abbreviation PK is strange.

Answer: Thank you for your comment, however, there is no consensus how to call the group of methyl ammonium lead halide perovskites. We can find in the literature different names: lead-halide perovskites (LHP), metal-halide perovskites (MHP), organic-inorganic halide perovskites (OHP) and many more. Therefore, we decided to use simple and short abbreviation PKs referring to APbX3 perovskites (where A = MA, Cs; X = Br, Cl in our case).

Reviewer: PK as an abbreviation refers to the crystal structure only, however, the authors work with a particular class of perovskites with are semiconductors with quite low bandgap. It is also misleading to use PK, because pK is well known abbreviation in chemistry, like pH and pOH. MHP covers all perovskites with metals and halides – the ones the authors work with.

13) Question 17 from my previous report

Page 4. “In PKs thin films, the defect states optical emission activity as well as absorption-induced photoconductivity is minimal” This sentence does make much sense to me. What is “emission activity”? “Activity” is a strange term to apply to defects. Photoconductivity, by its definition, is always induced by absorption. What do the authors really mean by this sentence?

Answer: Thank you for your comment. The sentence was rephrased "In PKs thin films, the defect states radiative emission activity, as well as absorption-induced photoconductivity, are low.

Reviewer: “Emission activity” is not the term, which is used in spectroscopy, this is slang. There is a “children activity” for example. Not activity of defects. “Optical activity” term exists; however, it is about completely different phenomena. The authors are talking about emission from defect states and photoexcitation of defects states. No new terms are needed in this discussion.

14) “Answer: Thank you for your comment. In the manuscript we state the dynamic range of PDS is 4 orders of absorption. However, we do not claim that it is significantly better than other methods. The advantage of the method in comparison with others is that it is sensitive to all absorption states. In PDS, no electron/hole pair collection (in case of FTPS) or radiative recombination (PL spectroscopy) is needed. Nevertheless, in comparison with transmission/reflection measurements, the sensitivity of PDS is much better (Ugur, et al.) especially in case of thick samples as ours.

Ugur, E.; Ledinský, M.; Allen, T. G.; Holovský, J.; Vlk, A.; De Wolf, S. Life on the Urbach Edge. J. Phys. Chem. Lett. 2022, 13 (33), 7702–7711. https://doi.org/10.1021/acs.jpclett.2c01812.”

Reviewer: I am confused. Ordinary absorption measurements also sensitive to all which does not light to go through the sample (non-radiative transitions + scattering, when the latter can be accounted for). What is the difference?

15) Question 23 from my previous report

23) p.9. Why is the heat going to propagate to the surface if, for example, the heat is uniformly generated through the whole thickness of the sample? Heat propagates anywhere is there is a gradient.

Answer: Thank you for your comment. As the sample is immersed in the liquid FC67, the heat will propagate from the sample to the colder liquid. This causes a large temperature gradient at the surface of the PK SC and thus the heat will propagate from the bulk towards the surface.

Reviewer: Is this the answer to the reviewer or this is explained in the revised version of the paper?

16) Question 24 from my previous report

24) P.10, top. Why is there a difference between surface to bulk and bulk – to- surface heat transfer?

Answer: Thank you for your question. The heat generated at the surface is transferred directly into the liquid. On the other hand, the heat generated in the bulk of the sample has to propagate towards the surface and then transfer into the liquid. This causes a time delay and thus phase shift between the signal generated at the surface and in the bulk of the sample.

Reviewer: Is this the answer to the reviewer or this is explained in the revised version of the paper?

15) Question 31 from my previous report

P.14. Why is the phase shift expected to be 45 degrees? Does it not depend on the thickness of the sample?

Answer: Thank you for this question. The thickness of our sample is approximately 2 mm. Therefore, the thickness dependence of the maximal phase shift considered as the sample is much thicker than the thermal diffusion length in this material. However, in general this phase shift varies with the thermal conductivity for different materials.

Reviewer: Is this the answer to the reviewer or this is explained in the revised version of the paper?

16) "Halide perovskites have extremely low thermal conductivity (k ≈ 0.37 – 0.51 W · m-1 · K-1)15–17 therefore, the thermal redistribution is limited to the volume near the front surface and the heat changes may be effectively detected."

Reviewer: Using relative terms like "extremely low" is not appropriate and may confuse the reader. The heat conductivity of MHPs is close to that of glass. Whether to consider this low or high – it depends. Here the authors can compare it with quartz (1.4W/m/K) as it is used as a substrate and with this special liquid used as the media.

Author's Response to Peer Review Comments:

Thank you for the review. We did our best to answer all the comments and make respective changes to our manuscript. Please find our answers in the attached file.

Reviewer(s)' Comments to Author:

Reviewer: 3

Recommendation: This paper represents a significant new contribution and should be published as is.

Comments:
All of the suggestions have been incorporated and reviewer comments have been addressed in the manuscript for the fulfilment of requisite quality of the journal. Hence, I recommend the publication of this work.

Additional Questions:
Urgency: High

Significance: Top 10%

Novelty: Top 10%

Scholarly Presentation: Top 10%

Is the paper likely to interest a substantial number of physical chemists, not just specialists working in the authors' area of research?: Yes

---

We would like to thank to the reviewer for all the work and time invested to improve our manuscript and for this positive review.

Reviewer 2:

Recommendation: This paper may be publishable, but major revision is needed; I would like to be invited to review any future revision.

Comments:
The authors did rather mild changes in the manuscript taking into account the number of questions asked. When a reviewer asks a question, it means that some changes (explanations) should be added to the text. In rare cases, when the answer to the question is obvious for most of the readers, just an answer to the reviewer is enough. I do not think that in the case of this particular manuscript the latter case is applicable. So, I am not happy with the authors approach to the revision. Still, my concern about the accuracy of the measurements and possible random and systematic errors remains due to absence of any reference sample. The authors need to address it. Note, metal-halide perovskites cannot be references due to their instability. See more details below.

We would like to thank to the reviewer for all the work and time invested to improve our manuscript. We have done all what was in our possibilities. We have to note, that PDS is well established method for low absorption measurement and its accuracy was proven by cited studies. Nevertheless, we have proven the accuracy for our specific case by three repeated measurements of the same sample, we have used several independent techniques and numerical calculations to validate our results. We are confined about our results, and we hope the results are also sufficient to convince the reviewer.

1) Page 8 “In order to obtain the intensity, the baseline caused by free carrier absorption was removed…” Why are the authors sure about the origin (free-carrier absorption) of this base line? Any measurement has an offset due to its technical realization. This base line may have nothing to do with the sample itself. As I have already

mentioned, the problem of this study is that the authors do not show any refence measurements and do not discuss errors and limitations of the technique.

Answer: Thank you for your comment. The increase of the absorption in the infrared region is observed for all semiconductors and is commonly related to the free-carrier absorption in semiconductors. We added references to our statement.

[1] FOX, Mark. Optical properties of solids. 2002.

[2] REMES, Zdenek, et al. Optical properties of SnO2: F films deposited by atmospheric pressure CVD. *Thin Solid Films*, 2009, 517.23: 6287-6289.)

2) Fig.1 a. The authors assigned peaks at 0.9 and 1,1 eV to real transitions. What is about the peak at 1.45 eV? Is it real or not? It is visible at most of the curves, but not at all of them. It is visible at the lowest and the highest frequency, but not at the middle ones. So, one needs to conclude that this peak is an artefact. In this case, why the other two peaks (0.9 and 1.1 eV) are not artefacts? All these peaks (0.9, 1.1 and 1.45) have about the same amplitude. There are many more "peaks" from 1.6 to 2 eV, are these peaks noise?

The authors must discuss this in detail, and clearly motivate what they consider as real signal and what not and explain why.

Answer: Thank you for your comment. The peak at 1.45 eV is discussed in the end of the article and identified as peak originated from surface defects. We added a sentence into this discussion, which points out that this peak is observed in the Fig. 1a as well. "… After the light exposure, an increase of absorption between 1.2 to 2.1 eV is observed, with two prominent bands at 1.45 and 1.60 eV. The same band at 1.45 eV was observed in the Fig. 1a … "

The two peaks at 0.9 and 1.1 were identified and independently confirmed by FTRI-transmission spectroscopy. This proves the origin of the peaks as well as validate the accuracy of PDS measurement on perovskite SC, please see Fig. S4 in SI:

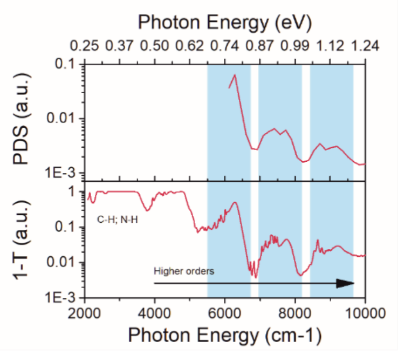

3) Fig.1 b. The figure cation does not tell what is presented in the panel b. The text is confusing, it should be extended and re-phrased. Also, the panel a shows the single crystal and the film, what is the sample shown in b) ? How one can possibly understand that the authors plot in Fig.1b something related to the "fit" function which was calculated for all frequencies? This is just one example of the "rebuses" the authors give to the readers. To understand the basics of a figure one does not need to read the whole paper.

Answer: Thank you for this comment. We added the information into the figure caption:

Figure 1. a) Frequency dependence of the normalized absorption spectra of $MAPbBr_3$ single crystal of a dimensions 5 x 5 x 2 mm and reference spectra of $MAPbBr_3$ thin film determined using PDS

method. b) Comparison of absorptance of $MAPbBr_3$ SC determined from the frequency dependence and thermal diffusion length dependence on modulation frequency. The inset of b) shows the part of the PDS spectra, from which the absorptance values at given frequency were taken.

4) Abstract "The identified absorption states are attributed to vibrational states and structural defects, and their influence on the non-radiative recombination probability is discussed"

Vibrational states do not exist in vacuum. What are these states? States of what? Molecules? Ions?

Answer: The origin of the vibrational states is discussed in the main text. We believe that the abstract is not the place for such discussion. However, we replaced "vibrational states" -> "MA+ vibrational states".

The detail description in the main manuscript text:

"That implies that these absorption states are directly associated with the internal vibrational states of MA+. Moreover, there is a constant energy difference of 0.185 eV between the detected peaks, which is approximately 1500 cm-1 and agrees with the energy of the vibrational modes of CH and NH bonds as measured by IR absorption spectroscopy."

5) "PKs thin films, the defect states radiative emission activity, as well as absorption-induced photoconductivity, are low."

What does defect activity mean? This is slang, not a scientific language. Absorption induced photoconductivity? Photoconductivity of MHP is very high as soon as you excite above the bandgap.

Answer: Thank you for your comment. We modified the sentence: "In MHPs thin films, the defect states radiative emission probability, as well as defect absorption-induced photoconductivity, are low."

6) Question 4 from my previous report

Absorption spectra and spectra of PTD should be compared for samples, where the sub-bandgap absorption is measurable by a standard absorption spectrometer.

Answer: Thank you for your comment. PDS itself is relatively common technique. We kindly refer you to our other work, where we compare several techniques for absorption measurements, E. Ugur et al.

Ugur, E.; Ledinský, M.; Allen, T. G.; Holovský, J.; Vlk, A.; De Wolf, S. Life on the Urbach Edge. J. Phys. Chem. Lett. 2022, 13 (33), 7702–7711. https://doi.org/10.1021/acs.jpclett.2c01812"

Reviewer: I looked at the paper suggested and did not find the answer to my question. I am asking about the particular sample, not any other one reported in another papers. As the authors can easily read from my questions, I simply do not fully trust the results they try to sell, and this the problem of the authors to convince me. So, my request is very simple: absorption spectra of the crystal measured using a traditional absorption spectrometer should be plotted together with the spectrum of the very same crystal obtained by PTD method.

Answer: Thank you for your comment. The PDS setup allow as to measure the transmission/reflection spectra at the same time. The comparison of the "standard" absorption measurement with PDS spectra in the sub-bandgap region is however not possible, since the "standard" absorption measurement accuracy is on the level of few %. This is the main reason, why

people are looking for methods with higher accuracy, in case of PDS is by two orders of magnitude better.

The second method, PTD, is not a spectral measurement. Single laser wavelength is used to excite the heat wave. Once the laser is exchanged for a white-light, the set-up is basically identical to PDS. So, with all the respect to the referee we do not see any sense in this comment. We need more detail comment on your idea, with references on the measurement and advantages of this kind of measurement over PDS, comment on the independent measurement by almost the same technique.

However, direct validation of PDS results may be found in the above referred manuscript, where one sample was measured by different techniques including PDS and standard spectrometer. All the measured spectra are in very good agreement. This clearly proves that there is no problem with absorption spectra measurement of these material in a form of thin films. In this manuscript we are discussing bulk single crystalline materials, where the effect of degradation on its properties is not as significant.

7) Question 5 from my previous report

There are no errors bars in the spectra. How does a reader know that the oscillations (jumping) of the signal are not just due to noise or systematic errors?

Answer: Thank you for your comment, we added the error bars for the phase measurements.

Reviewer: How were these bars (I do not see any bars. I see some shadowed regions) were estimated? How many spectra were used to estimate this “bars”?

Answer: Thank you for your comment. We do not see any problem in different graphical representation of “error bars”. Each point of every spectrum is measured several times (10-20x based on signal strength). The median is taken from those values, and these are the data points. The “error bars” presented are the standard deviation of the measurement.

We added this information into the Methods section: “In order to obtain the error of the measurement, each point is measured several times (10x-20x based on signal strength). The median is taken from those values, and these are the data points. The measurement errors are represented by the standard deviation.”

8) Fig. 4 b. The phase difference shows huge fluctuations over the spectrum. Are these fluctuations noise or not? Does only the offset (averaged over energy) have the meaning here? This must be mentioned in the fig caption.

Answer: Thank you for your comment. Yes, these fluctuations are noise. Lower the absorption signal is, the noisier it gets both in phase and absorption. We are providing the real data without smoothing them out. So, yes, one has to compare average over some energy interval and not at a single point. However, we do not see why this information should be in the figure caption, we see this point obvious for most readers.

9) Question 6 from my previous report

Is there any refence sample to compare? Also, a sample where absorption at the surface and bulk is different (I can easily image how I would make such a sample using a colour glass filter with an extra light absorbing layer deposited to the surface).

Answer: Thank you for the interesting idea. We did not perform such experiment. We believe that our examples of bismuth doping (bulk defects) and surface degradation prove together with presented calculations that suggested method works.

Reviewer: This is a bold statement which I totally disagree with. Surface degradation changes the morphology of the crystals and induce scattering. You cannot be sure that degradation occurs at the surface only. MHP cannot be used to test methods. This is because MHP are so strange materials, the materials which change their properties all the time. For example, why is the bismuth doping is a bulk doping? How can it be proved? It is just a hypothesis. The experiment I suggested is crucial (and easy) and I do not see why the authors cannot do it if they are sure about their technique.

Answer: Thank you for your comment. We would be happy to provide you with results of the experiment you suggest, however, we are not able to prepare such sample. We simply do not know how to prepare a thin film with absorption of only 0.1 % to mimic the situation we observe in our experiments. Additionally, colour filter is not the right substrate for this fundamental study, since the absorption is not well defined in the low absorption part. Absorption below 1% it is not relevant for the standard filter purpose, but we need to be sure about the absorption bellow 0.01%. We see the beauty of such an experiment, but unfortunately it is not easy to realize it. To our knowledge, the only bulk material measured by PDS in the transverse arrangement are the samples in our study.

The Bi doping is bulk doping as the bismuth is added to the solution before crystallization. The crystal at higher Bi concentration gets darker within its whole volume. This doping procedure is well known, and we use the recipe of Abdelhady et al. (ABDELHADY, Ahmed L., et al. Heterovalent dopant incorporation for bandgap and type engineering of perovskite crystals. *The journal of physical chemistry letters*, 2016, 7.2: 295-301.) We added this reference to our discussion: "… which confirms that the bismuth is uniformly distributed within the whole crystal volume. This is in agreement with the results of Abdelhady et al.[25] …"

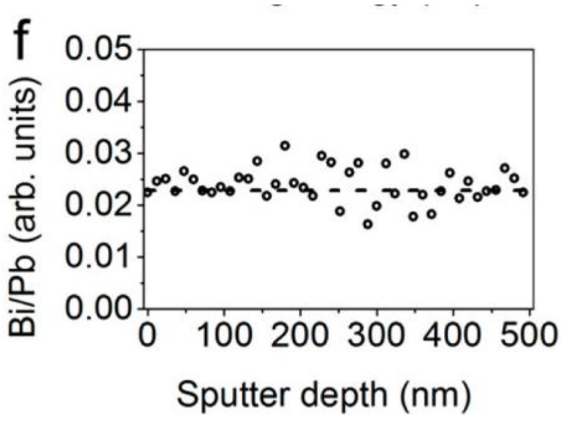

Figure 1: Sputter depth profile for the Bi/Pb ratio, Abdelhady et al.

10) Question 7 from my previous report

Basically, before one can discuss the defect states in perovskites and their location one need to prove that the method really works using a model sample with known properties.

Answer: Thank you for your comment. This comment was answered together with previous question. We have specifically chosen MAPbBr3 as a model material due to our extensive experience with this material.

Reviewer: I am dissatisfied with this answer. Perovskites are extremely difficult materials to work with. If the authors indeed have experience, as they claim, they know about it. I request the experiment with a dummy sample with different surface and bulk absorption (see above).

Answer: The absorption spectra of halide perovskite are well known, and we have the experience with its measurement by many different absorption techniques. The material stability used to be a problem for very first films prepared a decade ago. The stability of our perovskite SC is also confirmed by repeated experiment on the same sample, see below.

The problem with the dummy sample is in detail described above.

11) Question 8 from my previous report

Fig,5. The features in the absorption spectra are so sharp, that it is extremely hard to imagine that they are real. How does the same spectrum look like for another crystal of the same type? How does it look like if the setup is mistuned and tuned again? How does the signal look like for as piece of glass? The same concerns all other figures.

Answer: Thank you for your comment. The resolution in energy is 0.02 eV, therefore, those features may appear sharper than they really are. For example, for the peak at 1.44 eV, there is 5 points. To prevent random spikes appearing in the PDS spectra, each point of the spectra is acquired several times, and a median is taken. The PDS spectra and phase dependence of calibration sample are shown in the SI.

Reviewer: What are these "random spikes"? So, the method can give "random spikes", is it so? Why the peaks I see in each spectrum are also not random spikes? Or systematic spikes? It all comes back to the errors and convincing the readers that the signals are real, and clearly marking the signals which are obvious artefacts.

Answer: Thank you for your comment. We wrote: "To prevent random spikes appearing in the PDS spectra, each point of the spectra is acquired several times, and a median is taken." Of course, every method can give some random signal, for example due to the cosmic radiation hitting the detector and many other reasons. However, we do minimize the risk of getting random spike in the spectrum via the procedure described previously. Moreover, those peaks appearing in every spectrum (0.7, 0.9 and 1.1 eV) were compared with peaks measured using FTIR transmission, please see Fig. S4 in SI. This excludes them being systematic spikes.

12) Question 10 from my previous report

The authors need to make a detailed picture (cartoon) of the processes of heating/absorption, heat diffusion, refractive index change (where ? in the liquid? In the crystal?) and the role of the modulation frequency. On one side, the authors obviously try to explain all as good as possible in long and often related passages (not fully successfully, but still) and at the same time they used the figure of the setup, which was designed not by them and, therefore, it does not explain what they what to explain.

Answer: Thank you for your suggestion, we provided a modified picture in the SI

Reviewer: Details about the setup are given indeed. However, nothing understandable (orange-coloured

regions?) is given about the heat diffusion. This needs to be done. I was asking about the physical picture which would also explain the diffusion length and the frequency effect which is central for this study.

Answer: Thank you for your comment. I am sorry, but I do not know what your idea about the image is. How to draw picture of heat diffusion, or change of refractive index? We modified the picture again.

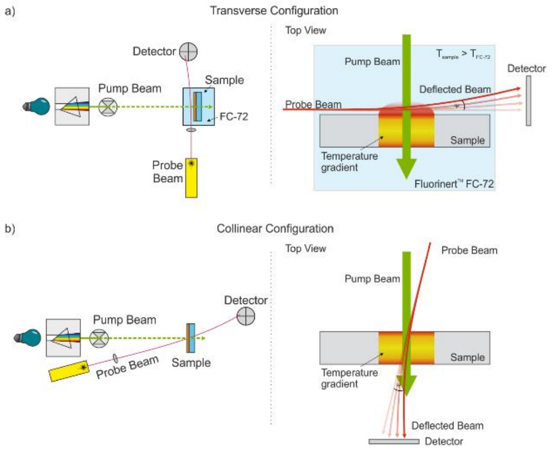

13) Question 11 from my previous report

How many crystals were measured? Several spectra of the same crystal measured at different alignments of the setup and spectra of different crystals of the same type should be shown.

Answer: Thank you for your comment: The measurement is repeatable. We have performed the same measurement on different samples and the results are in good agreement. It is not possible to repeat the measurement with different alignments as the measurement is done on optimized and calibrated setup.

Reviewer: First of all, I would like to see experiments measured on different samples (especially crystals). Second, of course, it is possible to measure at not identical conditions of the setup. This is how random errors originated form the setup alignment are estimated. It is very simple. You come to the lab next day. Tune the setup again - move all possible knobs out of the optimum and optimize the signal again. Place the sample in (the sample should be taken out and then set in again). All these manipulations are needed for any experimental technique to ensure that two measurements can be considered independent (or as independent as possible).

Answer: Thank you for your comment. Here, we provide comparison of three measurements of one MAPbBr3 single crystal. We measured these spectra after moving sample out of the optimal measurement position and repeat the alignment procedure. The repeatability of PDS measurement on perovskite SC is on very good level even for the low absorption sup-bandgap part and the phase values. We added this data into SI.

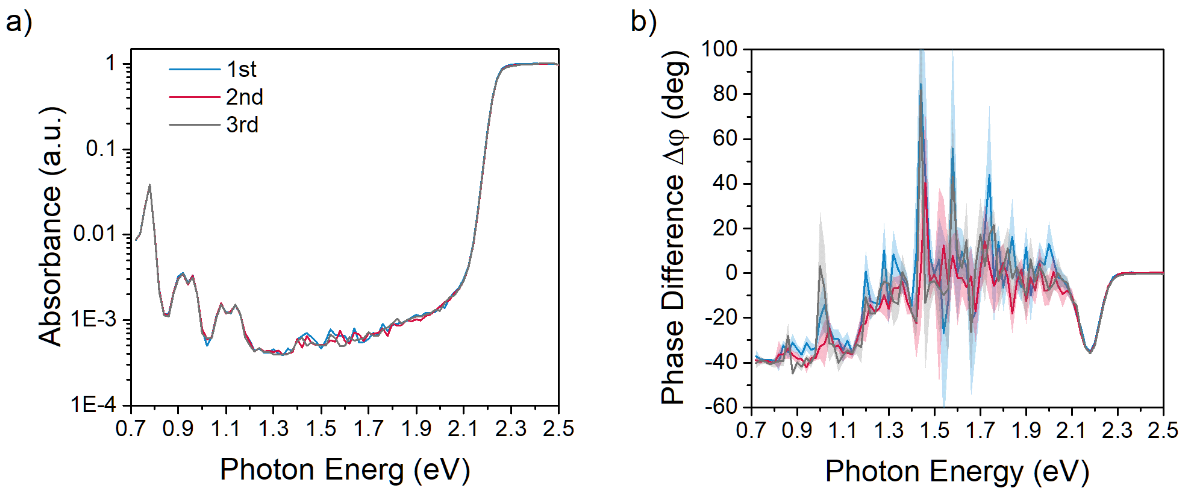

14) Question 16 from my previous report

Metal-halide perovskites are usually called MHP; abbreviation PK is strange.

Answer: Thank you for your comment, however, there is no consensus how to call the group of methyl ammonium lead halide perovskites. We can find in the literature different names: lead-halide perovskites (LHP), metal-halide perovskites (MHP), organic-inorganic halide perovskites (OHP) and many more. Therefore, we decided to use simple and short abbreviation PKs referring to APbX3 perovskites (where A = MA, Cs; X = Br, Cl in our case).

Reviewer: PK as an abbreviation refers to the crystal structure only, however, the authors work with a particular class of perovskites with are semiconductors with quite low bandgap. It is also misleading to use PK, because pK is well known abbreviation in chemistry, like pH and pOH. MHP covers all perovskites with metals and halides – the ones the authors work with.

Answer: Thank you for your comment. We do not understand why PK should be confused with pK. Nevertheless, we did change the abbreviation to MHP.

15) Question 17 from my previous report

Page 4. “In PKs thin films, the defect states optical emission activity as well as absorption-induced photoconductivity is minimal” This sentence does make much sense to me. What is “emission activity”? “Activity” is a strange term to apply to defects. Photoconductivity, by its definition, is always induced by absorption. What do the authors really mean by this sentence?

Answer: Thank you for your comment. The sentence was rephrased "In PKs thin films, the defect states radiative emission activity, as well as absorption-induced photoconductivity, are low.

Reviewer: “Emission activity” is not the term, which is used in spectroscopy, this is slang. There is a “children activity” for example. Not activity of defects. “Optical activity” term exists; however, it is about completely different phenomena. The authors are talking about emission from defect states and photoexcitation of defects states. No new terms are needed in this discussion.

Answer: Thank you for your comment. We modified the sentence: “In MHPs thin films, the defect states radiative emission probability, as well as defect absorption-induced photoconductivity, are low.”

16) “Answer: Thank you for your comment. In the manuscript we state the dynamic range of PDS is 4 orders of absorption. However, we do not claim that it is significantly better than other methods. The advantage of the method in comparison with others is that it is sensitive to all absorption states. In PDS, no electron/hole pair collection (in case of FTPS) or radiative recombination (PL spectroscopy) is needed. Nevertheless, in comparison with transmission/reflection measurements, the sensitivity of PDS is much better (Ugur, et al.) especially in case of thick samples as ours.

Ugur, E.; Ledinský, M.; Allen, T. G.; Holovský, J.; Vlk, A.; De Wolf, S. Life on the Urbach Edge. J. Phys. Chem. Lett. 2022, 13 (33), 7702–7711. https://doi.org/10.1021/acs.jpclett.2c01812.”

Reviewer: I am confused. Ordinary absorption measurements also sensitive to all which does not light to go through the sample (non-radiative transitions + scattering, when the latter can be accounted for). What is the difference?

Answer: Thank you for your comment. Yes, that is correct, transmission/reflection measurement is also sensitive to all transitions. However, as we stated in our previous answer, the sensitivity of this measurement is worser than for the PDS, see referred article (Ugur, et al.) and discussion above.

Ugur, E.; Ledinský, M.; Allen, T. G.; Holovský, J.; Vlk, A.; De Wolf, S. Life on the Urbach Edge. J. Phys. Chem. Lett. 2022, 13 (33), 7702–7711. https://doi.org/10.1021/acs.jpclett.2c01812.”

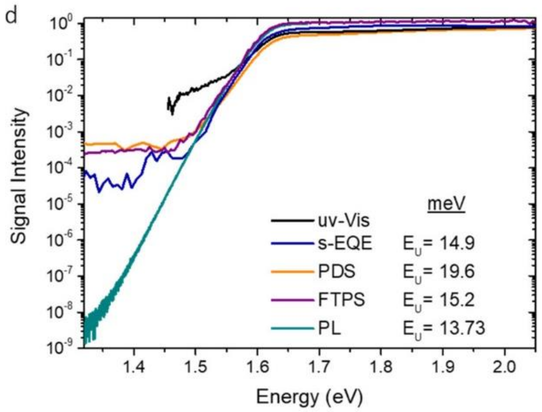

17) Question 23 from my previous report

23) p.9. Why is the heat going to propagate to the surface if, for example, the heat is uniformly generated through the whole thickness of the sample? Heat propagates anywhere is there is a gradient.

Answer: Thank you for your comment. As the sample is immersed in the liquid FC67, the heat will propagate from the sample to the colder liquid. This causes a large temperature gradient at the surface of the PK SC and thus the heat will propagate from the bulk towards the surface.

Reviewer: Is this the answer to the reviewer or this is explained in the revised version of the paper?

Answer: This is only the answer to the reviewer as it is a working principle of the method in transverse configuration, and it is explained in detailed in other referred publications [4-7].

18) Question 24 from my previous report

24) P.10, top. Why is there a difference between surface to bulk and bulk – to- surface heat transfer?

Answer: Thank you for your question. The heat generated at the surface is transferred directly into the liquid. On the other hand, the heat generated in the bulk of the sample has to propagate towards the surface and then transfer into the liquid. This causes a time delay and thus phase shift between the signal generated at the surface and in the bulk of the sample.

Reviewer: Is this the answer to the reviewer or this is explained in the revised version of the paper?

Answer: Thank you for your comment. The text was added into the manuscript. "… The heat generated directly at the surface is transferred immediately into the liquid and thus not retarded ($\Delta\phi$ = 0 deg) behind the pump beam. On the other hand, the heat generated in the bulk of the sample has to propagate towards the surface and then transfer into the liquid. This causes a time delay and thus increase in the phase shift ($\Delta\phi$ ~ 0 deg for strong absorption, $\Delta\phi$ < 0 deg for weak absorption). …"

19) Question 31 from my previous report

P.14. Why is the phase shift expected to be 45 degrees? Does it not depend on the thickness of the sample?

Answer: Thank you for this question. The thickness of our sample is approximately 2 mm. Therefore, the thickness dependence of the maximal phase shift considered as the sample is much thicker than the thermal diffusion length in this material. However, in general this phase shift varies with the thermal conductivity for different materials.

Reviewer: Is this the answer to the reviewer or this is explained in the revised version of the paper?

Answer: Thank you for your comment. The thicknesses of the samples were added into the Method section. The sentence was rephrased to "The phase difference at low energies should be constant, in this particular case at around 45 deg."

20) "Halide perovskites have extremely low thermal conductivity (k ≈ 0.37 – 0.51 W·m-1·K-1)15–17 therefore, the thermal redistribution is limited to the volume near the front surface and the heat changes may be effectively detected."

Reviewer: Using relative terms like "extremely low" is not appropriate and may confuse the reader. The heat conductivity of MHPs is close to that of glass. Whether to consider this low or high – it depends. Here the authors can compare it with quartz (1.4W/m/K) as it is used as a substrate and with this special liquid used as the media.

Answer: Thank you for this comment. We skipped the word "extremely".

Additional Questions:

Urgency: High

Significance: High

Novelty: Moderate

Scholarly Presentation: Moderate

Is the paper likely to interest a substantial number of physical chemists, not just specialists working in the authors' area of research?: Yes
